# Sea anemone Frizzled receptors play partially redundant roles in oral-aboral axis patterning

**DOI:** 10.1242/dev.200785

**Published:** 2022-10-12

**Authors:** Isabell Niedermoser, Tatiana Lebedeva, Grigory Genikhovich

**Affiliations:** ^1^ Department of Neurosciences and Developmental Biology, Faculty of Life Sciences, University of Vienna, Djerassiplatz 1, Vienna A-1030, Austria; ^2^ Vienna Doctoral School of Ecology and Evolution, University of Vienna, Vienna A-1030, Austria

**Keywords:** Cnidaria, *Nematostella*, Axial patterning, Gastrulation

## Abstract

Canonical Wnt (cWnt) signalling is involved in a plethora of basic developmental processes such as endomesoderm specification, gastrulation and patterning the main body axis. To activate the signal, Wnt ligands form complexes with LRP5/6 and Frizzled receptors, which leads to nuclear translocation of β-catenin and a transcriptional response. In Bilateria, the expression of different Frizzled genes is often partially overlapping, and their functions are known to be redundant in several developmental contexts. Here, we demonstrate that all four Frizzled receptors take part in the cWnt-mediated oral-aboral axis patterning in the cnidarian *Nematostella vectensis* but show partially redundant functions. However, we do not see evidence for their involvement in the specification of the endoderm – an earlier event likely relying on maternal intracellular β-catenin signalling components. Finally, we demonstrate that the main Wnt ligands crucial for the early oral-aboral patterning are Wnt1, Wnt3 and Wnt4. Comparison of our data with knowledge from other models suggests that distinct but overlapping expression domains and partial functional redundancy of cnidarian and bilaterian Frizzled genes may represent a shared ancestral trait.

## Teaser

**Summary:** In *Nematostella*, the ectoderm is patterned along the oral-aboral axis by Wnt/Fz/LRP5/6-mediated β-catenin signalling. In contrast, β-catenin-dependent endoderm specification does not seem to require Fz/LRP5/6.

## INTRODUCTION

Wnt ligands and their Frizzled (Fz) receptors are involved in multiple cellular signalling pathways, one of which leads to the nuclear accumulation of β-catenin and is termed the ‘canonical’ Wnt/β-catenin pathway or the cWnt pathway (MacDonald and He, 2012; van Amerongen and Nusse, 2009). In the ‘cWnt-off’ state, cytosolic β-catenin is continuously tagged for degradation by the ‘destruction complex’ containing APC, Axin, CK1α and GSK3β (Grainger and Willert, 2018), ubiquitylated by β-TrCP and degraded by the proteasome (Aberle et al., 1997). In the ‘Wnt-on’ state, a complex of Wnt, Fz and the co-receptor LRP5/6 forms at the membrane, which results in the sequestering of the destruction complex by Dishevelled, which, in turn, prevents tagging β-catenin for degradation (Gammons and Bienz, 2018; Willert et al., 1999). Non-tagged β-catenin accumulates in the cytosol and becomes translocated into the nucleus, where it displaces the transcriptional co-repressor Groucho and interacts with TCF to activate target genes (Flack et al., 2017). In addition to their role in cWnt signalling, which is characterized by the involvement of LRP5/6 and the nuclear translocation of β-catenin, Wnt ligands and Fz receptors are the starting points of multiple ‘non-canonical’ signalling pathways (Acebron and Niehrs, 2016; Croce and McClay, 2008; Garcia de Herreros and Duñach, 2019; Park et al., 2015; Semenov et al., 2007; Villarroel et al., 2020). In mammals, ten different Fz receptors that make up five families may demonstrate partially overlapping functions, and the effects of their individual or combined knockouts are usually attributed to a mixed action of the abnormal cWnt and non-canonical signalling (Fischer et al., 2007; Wang et al., 2016). Among the mammalian Fz receptors, only Fz4 appears to act exclusively in the cWnt pathway, while Fz3 and Fz6 seem to be exclusively involved in the Wnt/PCP pathway (Wang et al., 2016).

One of the ancestral roles of the β-catenin signaling is to define the gastrulation site, as well as to pattern the main body axis in animals – a feature that appears to be conserved across Metazoa. Localized expression of the Wnt signalling components along the main body axis has been documented in the earliest branching animal lineages such as ctenophores (Pang et al., 2010) and sponges (Adamska et al., 2010; Leininger et al., 2014). In Cnidaria, the bilaterian sister group, the role of the cWnt pathway in gastrulation and oral-aboral (OA) axis patterning has been confirmed by functional analyses (Kraus et al., 2016; Lebedeva et al., 2021; Leclère et al., 2016; Marlow et al., 2013; Momose et al., 2008; Momose and Houliston, 2007; Röttinger et al., 2012; Wikramanayake et al., 2003). Recently, we demonstrated that the regulatory logic of the β-catenin-dependent OA patterning in the sea anemone *Nematostella vectensis* and the posterior-anterior (PA) patterning of deuterostome Bilateria is highly similar, suggesting a common evolutionary origin of the OA and the PA axes (Darras et al., 2018, 2011; Kiecker and Niehrs, 2001; Lebedeva et al., 2021; Nordström et al., 2002). Although the way *Nematostella* interprets different intensities of β-catenin signal is largely understood (Kraus et al., 2016; Lebedeva et al., 2021), we still have very little idea about which Wnt ligands and which Fz receptors are involved in the cWnt-dependent axial patterning in this morphologically simple model organism. The complement of Wnt and Fz molecules in *Nematostella* is surprisingly large. It has representatives of 12 out of the 13 conserved bilaterian Wnt gene families, only lacking *Wnt9*, which has been lost in Cnidaria but is present in the earlier branching Ctenophora (Kusserow et al., 2005; Lee et al., 2006; Pang et al., 2010). *Nematostella* Wnt genes are expressed in staggered domains along the OA axis, with different Wnt sets transcribed in the ectoderm and in the endoderm (Kusserow et al., 2005; Lee et al., 2006). The *Nematostella* genome also harbours representatives of four out of five vertebrate Frizzled receptor families, Fz1/2/7 (Fz1 in the text below), Fz4, Fz5/8 (Fz5 in the text below) and Fz9/10 (Fz10 in the text below), and lacks only Fz3/6, which appears to be chordate specific (Bastin et al., 2015; Schenkelaars et al., 2015).

In this study, we asked which of the four Fz receptors and the many Wnt ligands are involved in the cWnt-dependent patterning of the oral-aboral axis in the *Nematostella* embryo. As the involvement of LRP5/6 is the hallmark of the cWnt signalling, we reasoned that analysing its loss-of-function phenotypes would tell us which parts of the OA patterning process are under cWnt control, thus facilitating the interpretation of the Fz loss-of-function data. We show that the knockdown of LRP5/6 suppresses the expression of the β-catenin-dependent oral and midbody genes, and expands aboral molecular identity without affecting endoderm specification. This results in a loss of the oral structures after gastrulation and a global expansion of the aboral/anterior molecular identity – a typical β-catenin loss-of-function phenotype. Individual knockdowns of the three orally expressed Fz genes do not affect oral marker gene expression. In contrast, dual- and triple-knockdowns of all possible Fz gene combinations partially phenocopy the LRP5/6 knockdown, while quadruple Fz gene knockdown replicates it at the molecular and morphological levels. These data suggest partial redundancy of the Fz receptors and involvement of all the *Nematostella* Fz receptors in cWnt-dependent OA patterning. We also demonstrate that Wnt1, Wnt3 and Wnt4 are the key Wnt ligands mediating OA patterning during early development.

## RESULTS

### Normal expression of the Fz and *LRP5/6* genes in *Nematostella*

We analysed temporal and spatial expression dynamics of Fz, *LRP5/6* and *LRP4/5/6-like* in *Nematostella* embryos and larvae by interrogating the NvERTx RNA-Seq database (Warner et al., 2018) and by performing whole-mount *in situ* hybridization. Transcriptomics data show that two out of four Fz genes, *Fz1* and *Fz5*, and *LRP5/6* are abundant in the unfertilized egg, and their expression is maintained at an approximately constant level. In contrast, the other two Fz genes, *Fz4* and *Fz10*, are zygotic and become activated around 8 h post-fertilization (hpf) (Fig. S1A). *LRP4/5/6-like* (Fig. S1B) is a weakly expressed gene that starts to be upregulated around 48 hpf; its expression becomes confined to the forming apical organ (Fig. S1C). Thus, we reasoned that *LRP4/5/6-like* is unlikely to be involved in cWnt signalling (at least not before 48 hpf) and did not consider it further.

**Fig. 1.**
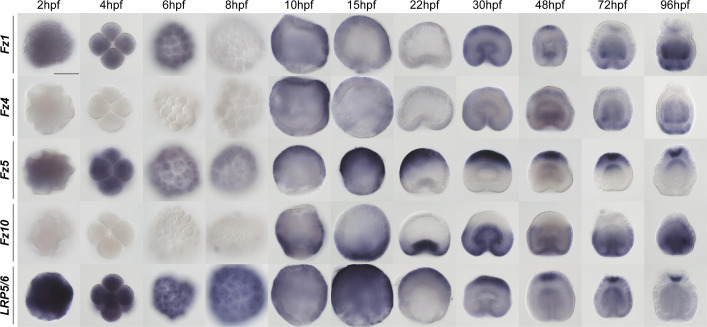
**Normal developmental expression of the Fz genes and *LRP5/6*.** From 10 hpf onwards, the animal/oral pole of the embryo is pointing downwards. *In situ* hybridization with digoxigenin-labelled RNA probes followed by anti-Dig-AP staining and NBT/BCIP detection. Scale bar: 100 µm.

*In situ* hybridization analysis of the *Fz1*, *Fz5* and *LRP5/6* (Fig. 1) show the initially ubiquitous distribution of the mRNA before 10 hpf, which then appears to be followed by the formation of a clearing in the expression that likely corresponds to the future pre-endodermal plate. At the same time, *Fz4* and *Fz10* expression starts to be detectable. As the development progresses, a second clearing in the *Fz10* expression domain appears on the putative aboral end, while *Fz5* expression becomes most prominent aborally (see also Lebedeva et al., 2021; Leclère et al., 2016; Röttinger et al., 2012; Wijesena et al., 2022). At the onset of gastrulation, *Fz1*, *Fz4* and *LRP5/6* are expressed ubiquitously; additionally, LRP5/6 is becoming ever more prominent in the aboral ectodermal domain. *Fz10* is expressed in the oral and midbody ectoderm, but it also starts to be strongly expressed in the invaginating endodermal plate. At late gastrula, a narrow clearing in *Fz1* expression starts to appear between the midbody ectoderm and the aboral ectoderm, and *Fz5* acquires an additional expression domain in the aboral endoderm. During planula development, *Fz1* expression forms an oral-to-aboral gradient with the maximum in the oral ectoderm and oral endoderm; however, *Fz1* transcript is also detectable in the apical organ. *Fz4* transcription forms a shallow oral-to-aboral gradient in both germ layers; however, in contrast to *Fz1*, *Fz4* is not expressed in the apical organ. *Fz5* is expressed in an aboral-to-oral gradient in both cell layers with the ectodermal expression fading out at the aboral/midbody boundary. Apical organ cells express *Fz5* particularly strongly, and there it is co-expressed with *Fz1*. Strong *Fz10* expression is detectable in the pharyngeal, oral and midbody ectoderm. Additionally, *Fz10* forms an oral-to-aboral gradient of expression in the endoderm. Finally, LRP5/6 is expressed ubiquitously; however, apical organ cells appear to produce much more *LRP5/6*, and a shallow aboral-to-oral gradient appears to exist in the endoderm (Fig. 1).

### LRP5/6 knockdown

**Fig. 2.**
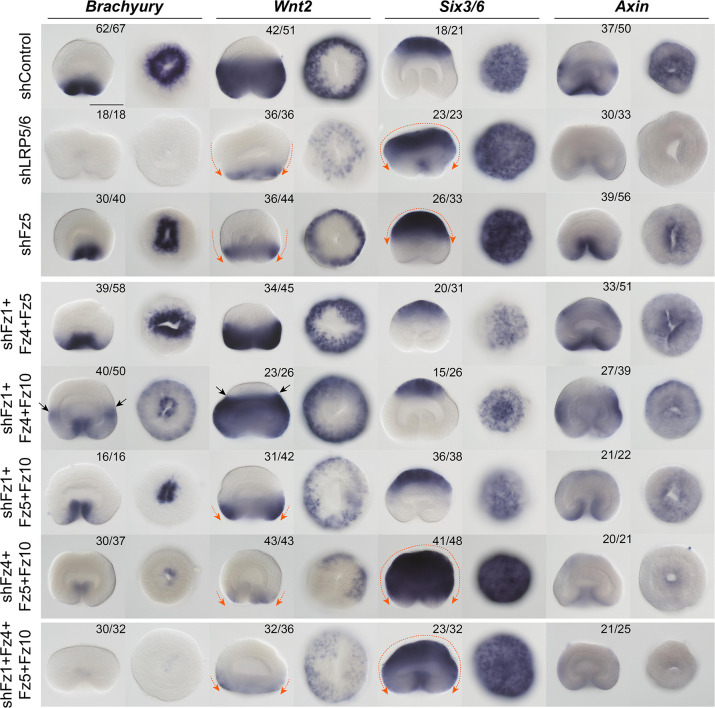
**Effects of RNAi-mediated KD of *LRP5/6* and *Fz5*, as well as triple and quadruple Fz gene KD combinations on the expression of the β-catenin-dependent markers of different axial domains in the 30 hpf late gastrula.** To keep the row labels readable, simultaneous RNAi of, for example, *Fz1*, *Fz5* and *Fz10* is marked as shFz4+Fz5+Fz10 rather than shFz4+shFz5+shFz10. The same labelling convention applies to all the other figures showing simultaneous KDs. Orange arrows indicate the direction of the drastic expression shifts. There is curious asymmetric expression of *Bra* and *Wnt2* upon *Fz4*+*Fz5*+*Fz10* RNAi, indicating possible abnormal feedback from the directive axis patterning mechanism. Black arrows indicate the ring of stronger *Bra* expression in the midbody and the aboral expansion of *Wnt2* domain, suggesting an ectopic enhancement of the β-catenin signalling. The numbers in the top right corners show the fraction of embryos demonstrating this phenotype. Scale bar: 100 µm. For each gene, lateral views (oral end down) are on the left and oral (or aboral in the case of *Six3/6*) views are on the right. *In situ* hybridization with digoxigenin-labelled RNA probes followed by anti-Dig-AP staining and NBT/BCIP detection.

**Fig. 3.**
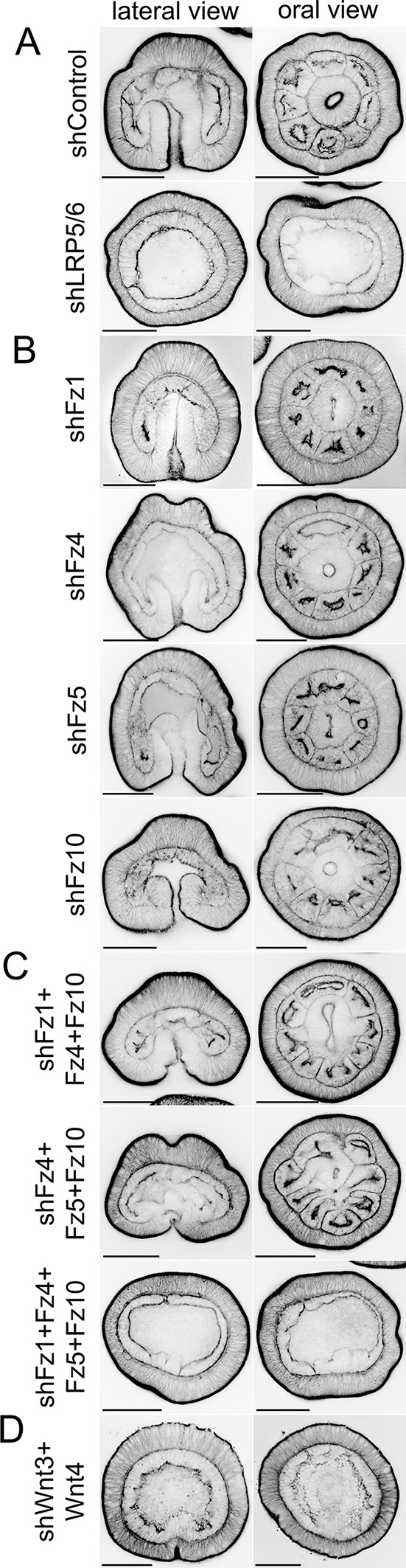
**Effects of the RNAi-mediated KD of *LRP5/6*, Fz genes and the *Wnt3/Wnt4* combination on the later development of the embryo.** (A-D) Effects of the KD of *LRP5/6* (A), of individual Fz genes (B), of triple and quadruple Fz gene KDs (C), and of the double KD of *Wnt3* and *Wnt4* (D). 4 dpf embryos are stained using phalloidin-AlexaFluor488 to visualize actin filaments. Scale bars: 100 µm. In the lateral views, the oral end points downwards.

**Fig. 4.**
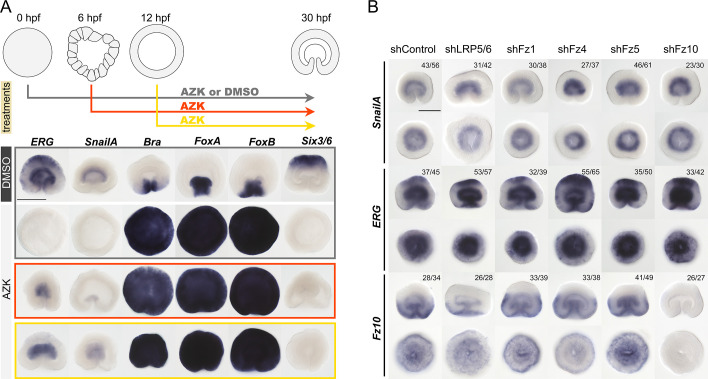
**Endoderm specification is an early event that does not seem to depend on Fz and LRP5/6.** (A) Identification of the time of endoderm specification. Lateral views of 30 hpf embryos, oral end downwards. (B) Endodermal marker expression is not affected by the KD of *LRP5/6* or by knockdown of individual Fz genes. The numbers in the top right corners show the fraction of embryos showing this phenotype. For each gene, lateral views (oral end down) are at the top and oral (or aboral in the case of *Six3/6*) views are at the bottom. All embryos are late gastrulae at 30 hpf. Scale bars: 100 µm. *In situ* hybridization with digoxigenin-labelled RNA probes followed by anti-Dig-AP staining and NBT/BCIP detection.

**Fig. 5.**
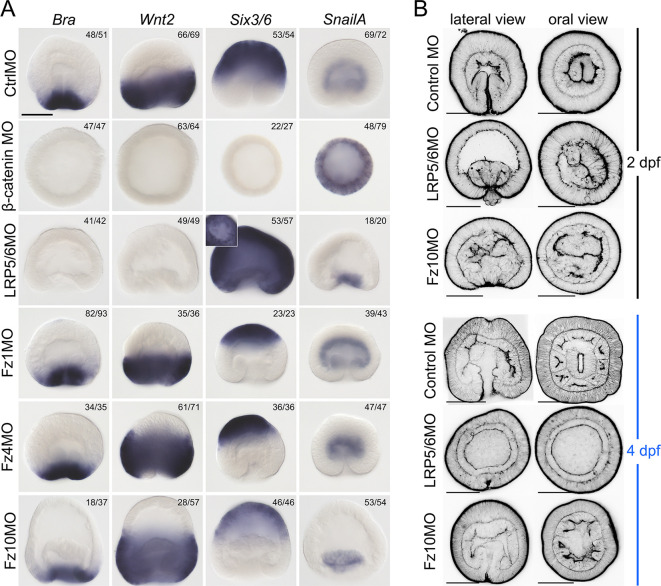
**Effect of the morpholino-mediated KD of *LRP5/6* and orally expressed Fz genes on the early development of *Nematostella*.** (A) Effect of the knockdowns on the expression of the markers of the distinct axial domains in the ectoderm and on the expression of the endodermal marker *SnailA*. *In situ* hybridization with digoxigenin-labelled RNA probes followed by anti-Dig-AP staining and NBT/BCIP detection. All embryos are fixed at 30 hpf. The numbers in the top right corners show the fractions of embryos demonstrating this phenotype. Scale bar: 100 µm. Lateral views, oral end downwards. The inset image of an oral view of the LRP5/6 morphant stained for *Six3/6* shows that the pre-endodermal plate does not express *Six3/6*. (B) Effects of the LRP5/6 and Fz10 morpholino KDs on the later development of the embryos. Phalloidin staining of the 2 dpf and 4 dpf planulae. Scale bars: 100 µm.

To assess the role of *LRP5/6*, we performed shRNA-mediated knockdowns (KDs, Fig. S2A-C) and characterized their effect on marker gene expression. We used *Brachyury* (*Bra*), *Wnt2* and *Six3/6* as markers of the oral, midbody and aboral domains, respectively (Lebedeva et al., 2021; Sinigaglia et al., 2013), *Axin* as a β-catenin signalling target gene with broader expression (Kraus et al., 2016; Lebedeva et al., 2021), as well as several additional markers for specific areas in the embryo. Notably, the midbody marker *Wnt2* is also positively regulated by β-catenin signalling but it is suppressed orally by Bra (Lebedeva et al., 2021). At the late gastrula stage (30 hpf), *LRP5/6* RNAi resulted in a strong suppression of the oral markers *Bra*, *FoxA* and *FoxB*, as well as *Axin* (Fig. 2, Fig. S3). *Wnt2* was reduced and only detectable in the oral domain, while *Six3/6* strongly expanded orally and acquired an additional area of expression in the pharyngeal ectoderm (Fig. 2). The suppression of the oral ectodermal and the expansion of the aboral ectodermal domain signature into the oral ectodermal territory persisted into later developmental stages, even though *LRP5/6* expression was re-established by 3 days post fertilization (dpf) (Fig. S4). Despite normal gastrulation, oral and pharyngeal structures were later lost, and by 4 dpf all LRP5/6 RNAi embryos resembled diploblastic spheres (Fig. 3A). In summary, LRP5/6 RNAi phenocopied the outcome of dominant-negative *Tcf* (*dnTcf*) mRNA overexpression (Röttinger et al., 2012), and was strikingly similar to the effect of the combined KD of *Bra*, *Lmx*, *FoxA* and *FoxB* – the four β-catenin-dependent transcription factors determining the oral molecular identity of the embryo (Lebedeva et al., 2021). Thus, LRP5/6 RNAi resulted in a typical β-catenin loss-of-function phenotype (Leclère et al., 2016), apart from the obvious fact that the embryos gastrulated normally, which was also the case in *dnTcf* mRNA-injected embryos (Röttinger et al., 2012) but, curiously, not in β-catenin morphants (Leclère et al., 2016) or in embryos subjected to shRNA-mediated β-catenin RNAi (Karabulut et al., 2019). Unlike *LRP5/6* RNAi and *dnTcf* overexpression, β-catenin morpholino injection resulted in a complete suppression of the oral, midbody and aboral ectoderm markers, and in a ubiquitous upregulation of the endodermal marker *SnailA* (Leclère et al., 2016). In contrast, pharmacological activation of β-catenin signalling with azakenpaullone (AZK) starting at fertilization also blocked gastrulation; however, in this case, *SnailA* expression was abolished, and oral ectoderm markers were expressed ubiquitously instead (Leclère et al., 2016, see also Fig. 4A). Curiously, endodermal marker expression, as well as the gastrulation process, was not affected by AZK treatment if the treatment started after 6 hpf (Fig. 4A), which corresponds to the reported time of the activation of the zygotic genome (Helm et al., 2013). This suggests that endoderm specification probably relies on maternally deposited mRNA and proteins, and occurs before 6 hpf, and that, once specified, the endoderm becomes insensitive to modulations in β-catenin signalling at least until late gastrula stage. Moreover, normal gastrulation and endodermal marker gene expression in shLRP5/6 embryos (Fig. 4B) raises the possibility that endoderm specification and the gastrulation movements, although obviously β-catenin dependent, may not require Wnt/Fz/LRP5/6-mediated signalling. To address this in more detail, we first asked how soon the effect of LRP5/6 knockdown started to manifest itself after the RNAi. Despite clear *LRP5/6* suppression as early as 6 hpf, the effect of *LRP5/6* RNAi on the sensitive β-catenin signalling target *Bra* was not apparent at 10 hpf, and only became observable at late blastula (18 hpf) stage (Fig. S5). As this comparatively late manifestation of the LRP5/6 RNAi effect, rather than endoderm specification and invagination being Wnt/Fz/LRP5/6-independent, may be the reason for the difference between the morpholino-mediated β-catenin KD and the RNAi-mediated *LRP5/6* KD, we repeated *LRP5/6* KD using a translation-blocking morpholino (MO, Fig. S2C). By 30 hpf (late gastrula stage in controls), LRP5/6 MO injection resulted in a phenotype similar to that of LRP5/6 RNAi, although more pronounced: not only *Bra*, but also *Wnt2* expression was abolished, and *Six3/6* was expanded throughout the whole ectoderm. In contrast to LRP5/6 RNAi, gastrulation was delayed in the morphants; nevertheless, as for RNAi, the specification of the *SnailA*-positive, *Six3/6*-negative pre-endodermal plate took place normally (Fig. 5A). By 48 hpf, the LRP5/6 MO-injected embryos remained arrested in gastrulation, demonstrating a miniature blastopore lip and a slightly submerged endoderm (Fig. 5B). By 4 dpf, LRP5/6 morphants displayed the same ‘bi-layered aboralized sphere’ phenotypes as the LRP5/6 RNAi embryos (Figs 3A and 5B). Both RNAi- and MO-mediated knockdown clearly show that LRP5/6 is required for the cWnt-mediated patterning of the ectoderm in *Nematostella*. The conspicuous lack of endodermal mesenteries in the 4 dpf LRP5/6 RNAi and morphant embryos is a clear sign of the disrupted BMP signalling resulting in the loss of the second, ‘directive’ body axis (Genikhovich et al., 2015; Leclère and Rentzsch, 2014). Previously, we have demonstrated that β-catenin is required for the onset of the expression of *BMP2/4* and *Chordin* – the core components of the BMP signalling network in *Nematostella* (Genikhovich et al., 2015; Kirillova et al., 2018; Saina et al., 2009). Surprisingly, upon LRP5/6 RNAi, the directive axis is formed, but later lost, as evidenced by *Chordin* expression, which is initially normal and bilaterally symmetric at the gastrula stage, but disappears by 3 dpf (Fig. S6). In summary, we conclude that *LRP5/6* is required for the β-catenin-dependent patterning of the ectoderm along the OA axis, and for the maintenance of the directive axis, but we do not find evidence of its involvement in the specification of the endoderm.

### Knockdown of Fz receptors

In contrast to *Fz5* RNAi, which reproduced the Fz5 morpholino knockdown phenotype published earlier (Leclère et al., 2016; this paper), individual RNAi of *Fz1*, *Fz4* and *Fz10* did not result in changes in the *Bra*, *Wnt2*, *Six3/6* and *Axin* expression (Fig. S7). The *Fz5* RNAi phenotype was similar to that of *LRP5/6* RNAi, with the aboral, *Six3/6*-expressing domain expanded, and the midbody *Wnt2*-expressing domain constricted towards the oral pole (Fig. 2). However, in contrast to *LRP5/6* RNAi, oral markers *Bra, FoxA* and *FoxB* were not affected by *Fz5* RNAi, and only the midbody expression of *Axin* was suppressed, while oral expression was retained (Fig. 2, Fig. S3). Endodermal expression of *SnailA*, *ERG* and *Fz10* was also not affected by any of the Fz RNAi knockdowns, except for *Fz10* expression, which, naturally, was abolished upon *Fz10* RNAi (Fig. 4B). Individual Fz gene RNAi did not lead to significant morphological defects apart from a slight gastrulation delay in *Fz1* and *Fz10* RNAi, and a previously reported slight shortening of the OA axis in *Fz5* RNAi (Leclère et al., 2016). By 4 dpf, the KD embryos developed eight normal mesenteries (Fig. 3B).

Surprisingly, these results contradicted the recently published *Fz1* and *Fz10* KD phenotypes (Wijesena et al., 2022). In this paper, the authors stated that overexpression of the dominant-negative form of *Fz1* (*dnFz1*) caused oral expansion of *Fz5*, suppression of *FoxA* in the blastopore lip, and the loss of the endodermal expression of *SnailA* and *Fz10* without interfering with the gastrulation process. In contrast, their Fz10 morpholino injection suppressed endoderm invagination without affecting *SnailA*. This latter result was somewhat surprising, as the disappearance of *Fz10* expression Wijesena et al. observed upon *Fz1* KD did not lead to a gastrulation failure. These results led the authors to conclude that Fz1 was controlling the cWnt-dependent specification of the endoderm, while Fz10 was regulating the non-canonical Wnt-dependent endoderm invagination (Wijesena et al., 2022).

Remembering the more pronounced effect of morpholino-mediated *LRP5/6* KD in comparison with RNAi, we repeated individual *Fz1*, *Fz4* and *Fz10* KD using the Fz1MO, Fz4MO and Fz10MO (Fig. 5, Fig. S2D). Similar to the RNAi result, Fz1MO and Fz4MO injection did not lead to changes in the expression of the oral, midbody, aboral and endodermal markers (Fig. 5A). In our hands, the overexpression of *dnFz1-mCherry* mRNA also did not cause any change in *Bra*, *Wnt2*, *Six3/6* and *SnailA* expression (Fig. S8), similar to the *Fz1* RNAi and Fz1MO KD. In contrast, Fz10 morpholino injection led to a delay in gastrulation without affecting *Bra*, *Wnt2*, *Six3/6* and *SnailA* expression (Fig. 5A). By 48 hpf, Fz10MO morphants completed invagination, although their endoderm still looked irregular and they had open blastopores (Fig. 5B). Their morphology mostly normalized by 96 hpf, with the only deviation being the lower number of mesenteries suggesting developmental delay or, potentially, problems with integrating the oral-aboral and the directive axis patterning (Fig. 5B). Thus, it is likely that *Fz10* plays a role in regulating gastrulation; however, the similarity of the effect of Fz10 and LRP5/6 morpholino KD on the overall morphology of the gastrula raises the possibility that the gastrulation delay may be caused by the cWnt signalling-related defect. The proposed role of Fz10 in mediating non-canonical Wnt signalling cannot be excluded and has to be directly assessed in the future; however, we do not find clear support for the ‘Fz1 for cWnt and endoderm specification versus Fz10 for non-canonical Wnt signalling and endoderm invagination’ distinction proposed previously (Wijesena et al., 2022).

As individual RNAi of the orally expressed Fz genes did not elicit an effect, we presumed that they might be partially or completely redundant at the gastrula stage, and performed simultaneous RNAi of all possible combinations of two, three or four Frizzleds. Double Fz gene knockdowns showed effects on marker genes only if shFz5 was in the mix, and recapitulated the individual *Fz5* KD (Fig. S7). In triple RNAi, a β-catenin loss-of-function phenotype similar to the *LRP5/6* RNAi started to emerge in some cases, most notably in the *Fz4*+*Fz5*+*Fz10* combination (Fig. 2). Simultaneous RNAi of *Fz1*+*Fz4*+*Fz10* resulted in a curious phenotype, which we are currently unable to explain: expression of *Bra* and *Axin* at the oral end of the gastrula became weaker, and a narrow ring of relatively strong *Bra* expression and a wider ring of strong *Axin* expression appeared in the midbody of the gastrula, suggesting stronger than usual β-catenin signalling in this area. This occurred concomitantly with the aboral expansion of the *Wnt2* domain and reduction of the *Six3/6* domain. *Wnt2* expression in this case was strongest in an area located between the Bra-expressing ring in the midbody and the diminished *Six3/6* expression domain (Fig. 2). In spite of the prominent effects at the gastrula stage, triple Fz gene RNAi embryos formed eight mesenteries by 4 dpf, although the mesenteries in the *shFz4*+*Fz5*+*Fz10* combination always looked somewhat irregular (Fig. 3C). Finally, quadruple RNAi of all four Fz receptors phenocopied LRP5/6 RNAi at the molecular as well as at the morphological level (Figs 2 and 3A,C). Taken together, we show that three orally expressed Fz receptors play a partially redundant function in the OA axis patterning of the *Nematostella* gastrula. The fact that only combined RNAi of all four Fz receptors phenocopies the LRP5/6 knockdown at the molecular and morphological level hints towards the involvement of all *Nematostella* Fz proteins in the LRP5/6-mediated cWnt signalling. We do not find evidence that endoderm specification depends on LRP5/6/Fz-mediated β-catenin signalling.

### Knockdown of Wnt ligands

**Fig. 6.**
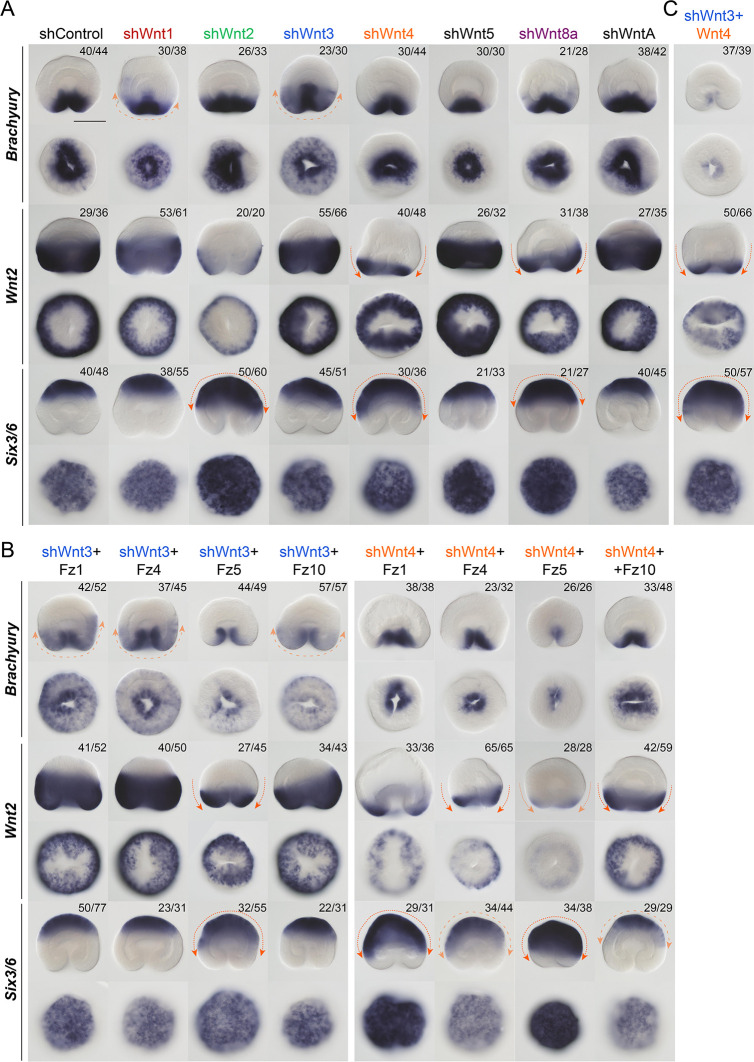
**Effect of the KD of Wnt genes on the expression of the oral, midbody and aboral ectoderm markers in the 30 hpf gastrulae.** (A) KDs of individual Wnt genes. (B) Simultaneous KDs of *Wnt3* or *Wnt4* with the individual Fz receptor genes. (C) Simultaneous KDs of *Wnt3* and *Wnt4*. Orange arrows indicate the direction of the particularly drastic expression shifts. The numbers in the top right corners show the fractions of embryos demonstrating this phenotype. Scale bar: 100 µm. For each gene, lateral views (oral end downwards) are at the top and oral (or aboral in the case of *Six3/6*) views are at the bottom. *In situ* hybridization with digoxigenin-labelled RNA probes followed by anti-Dig-AP staining and NBT/BCIP detection.

Wnt genes of *Nematostella* are expressed in staggered domains along the OA axis (Kusserow et al., 2005; Lee et al., 2006); however, their individual roles in OA patterning are still unclear. We have shown previously that co-expression of two Wnt genes, *Wnt1* and *Wnt3*, was sufficient to convey axial organizer capacity to any area of the *Nematostella* gastrula ectoderm, while other early Wnt ligands failed to elicit this effect (Kirillova et al., 2018; Kraus et al., 2016). However, even for *Wnt1* and *Wnt3*, the possible role in axial patterning was not analysed. In order to achieve some indication of which Wnt ligands might be involved in transmitting the signals patterning the *Nematostella* ectoderm along the OA axis, we analysed the loss-of-function phenotypes of all the Wnt genes expressed in the early embryo of *Nematostella*. The following Wnt genes are active in the embryo at or before gastrula stage: *Wnt1*, *Wnt2*, *Wnt3*, *Wnt4*, *Wnt5*, *Wnt8a* and *WntA* (Fig. S9). RNAi of *Wnt5*, which was not very efficient with both shRNAs we used, and *WntA* did not elicit any noticeable effect on the expression of *Bra*, *Wnt2* and *Six3/6* in the gastrula. RNAi of the orally expressed *Wnt1* and, even more prominently, of *Wnt3* resulted in a reduction of the expression of the oral marker *Bra* and its expansion to the bottom of the pharynx (Fig. 6A, Fig. S10) – a phenotype similar to the KD effect of one of the four key regulators of the oral molecular identity: *FoxB* (Lebedeva et al., 2021). RNAi of *Wnt2* and *Wnt8a*, which are normally expressed in the midbody domain, resulted in the moderate oral expansion of the aboral marker *Six3/6*, while the KD of the orally expressed *Wnt4* led to a strong aboralization of the embryo comparable with the effect of *Fz5* KD (Fig. 6A). None of the RNAi-mediated *Wnt* KDs affected *SnailA* expression (data not shown) or gastrulation. In spite of the oral-aboral marker expression changes we observed at 30 hpf in several individual Wnt KDs, the embryos appeared to have regulated their development by 4 dpf, building normal pharynges and mesenteries (Fig. S11).

Next, we tested whether concomitant knockdowns of the individual Fz receptors would lead to a synergistic effect with any of the Wnt genes, which showed an oral-aboral phenotype at 30 hpf upon individual KDs (Fig. 6B, Fig. S12). Simultaneous KD of *Wnt3* with individual Fz receptor genes showed a more prominent reduction in the expression of the oral marker *Bra* than *Wnt3* KD alone in all combinations. However, this effect was strongest in the shWnt3+Fz10 and the shWnt3+Fz5 combinations. A similar, although slightly weaker, effect was observed in the shWnt1+Fz combinations (Fig. S12). When shWnt4+Fz combinations were tested, the effects were even more noticeable. Oral expression of *Bra* was reduced in all shWnt4+Fz combinations in comparison with *Wnt4* RNAi alone. The aboralization of the embryo characteristic for the *Wnt4* KD was observed in all combinations; however, simultaneous KD of *Wnt4* and *Fz5* resulted in a more extensive aboralization than that observed upon individual KDs of *Wnt4* or *Fz5*, suggesting Fz5 as a highly probable interaction partner for Wnt4 – a hypothesis that can be tested by biochemical analyses in the future. Other shWnt+Fz double KDs did not lead to a noticeable synergistic effect (Fig. S12). Finally, we tested the result of the double KD of different Wnt genes. The strongest synergistic effect was observed in the shWnt3+Wnt4 and (to a slightly lesser degree) in the shWnt1+Wnt4 combination. Similar to the *LRP5/6* KD and the combined KD of all Fz receptors, shWnt3+Wnt4, as well as shWnt1+Wnt4, resulted in a strong aboralization of the gastrula, and loss of the oral structures and mesenteries by 4 dpf (Figs 3D and 6C, Figs S13, S14). Thus, we conclude that Wnt1, Wnt3 and Wnt4 are required for the LRP5/6/Fz-dependent OA patterning, and for the maintenance of the directive axis in *Nematostella*.

## DISCUSSION

The role of Wnt/Fz-mediated signalling in development and disease is difficult to overestimate; however, the variety of signalling pathways that may be activated by a Wnt-Fz interaction makes such investigation highly challenging. The initial hope that a multitude of vertebrate Fz receptors and a corresponding multitude of Wnt ligands would fall into an orderly system of signalling partnerships was not supported by the data. Phylogenetic analyses showed that the large diversity of the conserved Wnt gene families in Planulozoa (Cnidaria+Bilateria) is much more ancient than the Fz gene diversity found in vertebrates (Kusserow et al., 2005). Instead, non-vertebrate planulozoans normally have four Fz genes: *Fz1/2/7*, *Fz4*, *Fz5/8* and *Fz9/10*, which have to cope with all the various Wnt ligands (Bastin et al., 2015; Janssen et al., 2015; Qian et al., 2013; Robert et al., 2014; Wijesena et al., 2022). Work on bilaterian – mostly vertebrate – models demonstrated partial redundancy of Fz receptors, as well as the involvement of the same receptors in both the cWnt and the non-canonical Wnt signalling (Bhat, 1998; Dong et al., 2018; Fischer et al., 2007; Voloshanenko et al., 2017; Wang et al., 2016; Yu et al., 2012).

One of the Wnt-mediated signalling pathways, the cWnt or Wnt/β-catenin pathway, appears to be the oldest axial patterning system present in animals. cWnt pathway involvement in the patterning of the PA axis of Bilateria and the OA axis of Cnidaria has been convincingly demonstrated functionally during the past 30 years (Darras et al., 2018, 2011; Fu et al., 2012; Kiecker and Niehrs, 2001; Kraus et al., 2016; Lebedeva et al., 2021; Marlow et al., 2013; McCauley et al., 2015; Nordström et al., 2002; Prühs et al., 2017; Range et al., 2013), and expression data suggest that cWnt may also be responsible for axial patterning in the earlier branching ctenophores and sponges (Leininger et al., 2014; Pang et al., 2010). Another ancestral developmental function of β-catenin appears to be the definition of the endomesodermal and, subsequently, the endodermal domain during germ layer specification in Bilateria and Cnidaria (Henry et al., 2008; Leclère et al., 2016; Lhomond et al., 2012; Logan et al., 1999; Martín-Durán et al., 2016; Momose et al., 2008; Momose and Houliston, 2007; Wikramanayake et al., 2003). Among cnidarians, the role of Fz-mediated signalling in gastrulation and OA patterning has been addressed in a hydroid *Clytia hemisphaerica*. There, two Fz mRNAs, *CheFz1* (*Fz1/2/7* ortholog) and *ChFz3* (*Fz9/10* ortholog), are maternally localized to the animal and the vegetal hemispheres of the egg, respectively, and appear to have opposing functions. *CheFz1* KD results in a delayed endoderm formation and suppression of the animal/oral marker gene expression, while *CheFz3* KD leads to the oralization of the embryo, abolishes vegetal/aboral marker genes and accelerates the ingression of the endodermal cells (Momose and Houliston, 2007). *CheFz1* is also reported to be involved in the Strabismus/Dishevelled-mediated embryo elongation in *Clytia*, suggesting that CheFz1 is active in the cWnt as well as in the Wnt/PCP pathways (Momose et al., 2012). *CheWnt3* (*Wnt3* ortholog), the mRNA of which is maternally localized to the animal pole, appears to be the key ligand responsible for the oralization, likely by signalling via CheFz1 (Momose et al., 2008). This mode of regulation, however, does not recapitulate the situation we observed in the anthozoan cnidarian model *Nematostella vectensis*. In *Nematostella*, *Fz1*, *Fz5* and *LRP5/6* mRNAs are maternally deposited; however, these mRNAs are evenly distributed throughout the egg. Fz gene expression during early development is in partially overlapping domains, and it roughly recapitulates the expression of Fz genes in sea urchin embryos of comparable stages (Robert et al., 2014). With the possible expression of *Wnt5*, which shows some maternal transcript (Fig. S9), *Nematostella* Wnt genes, *Fz4* and *Fz10* are zygotically expressed. Proteomics data indicate that, among the four Fz receptors, LRP5/6 and all Wnt ligands, only Fz5 protein is detectable in the *Nematostella* egg, early cleavage and blastula stage embryos (Levitan et al., 2015). Our AZK treatment experiments suggest that β-catenin-dependent specification of the future pre-endodermal plate is an early event that occurs before the onset of the zygotic transcription around 6 hpf, and is thus likely to rely on maternally deposited molecules. We observed normal endoderm invagination, normal expression of the endodermal markers *SnailA* and *ERG* (Fig. 4B), which are negatively controlled by β-catenin, and the lack of the expression of the β-catenin signalling targets such as *Bra* in the endoderm of the embryos treated with AZK after 6 hpf (Lebedeva et al., 2021). This indicates that, after being specified, the future endoderm becomes insensitive to the modulation of the β-catenin signalling intensity. Moreover, normal endoderm specification upon RNAi and morpholino knockdowns of the maternally deposited *LRP5/6*, *Fz1* and *Fz5* suggest that this process may not require Fz/LRP5/6-mediated signalling but relies on the cytoplasmic components of the β-catenin signalling pathway. Because currently we cannot fully exclude the possibility that some LRP5/6 and Wnt protein remained undetected in all the proteomic datasets (Levitan et al., 2015) or that their translation from maternal mRNA was not sufficiently suppressed in our KDs, additional genetic work will be required. In the future, generation and incrossing of the β-catenin^wt/−^ and LRP5/6^wt/−^ knockout lines will allow us to definitively answer the question of whether or not endoderm specification relies on maternal β-catenin and is LRP5/6 independent, as our data currently seem to suggest. The gastrulation delay in LRP5/6 morphants also indicates the likely involvement of LRP5/6-mediated β-catenin signalling in the process of gastrulation.

In echinoderms, the early β-catenin-dependent specification of the endomesodermal domain is followed by the segregation of the endoderm from the mesoderm, and the subsequent Wnt-dependent PA patterning. In the endoderm, β-catenin signalling remains strong, while in the mesoderm β-catenin signalling becomes suppressed (Lhomond et al., 2012; Logan et al., 1999; McCauley et al., 2015; McClay et al., 2021; Range et al., 2013; Sun et al., 2021; Wikramanayake et al., 1998, 2004). A similar sequence of events – the early β-catenin-dependent definition of the future endodermal domain, the formation of the boundary between the β-catenin-sensitive future oral ectoderm and the β-catenin-insensitive future endoderm – and the subsequent Wnt-dependent OA patterning of the ectoderm also occurs in *Nematostella*, and these events seem to follow the same regulatory logic as described for the sea urchin. Recently, we described the regulatory principle underlying β-catenin-dependent OA patterning of the ectoderm in *Nematostella*, which leads to the subdivision of the ectoderm into oral, midbody and aboral domains (Lebedeva et al., 2021). This subdivision happens as follows. A number of transcription factor-coding genes, the expression of which is positively regulated by β-catenin signalling, start to be expressed in the oral hemisphere of the *Nematostella* embryo. Their expression resolves into specific domains along the oral-aboral axis because some of these genes, which are expressed more orally, encode transcriptional repressors acting on the genes, which are expressed more aborally. This creates the two main molecular boundaries of the early embryo of *Nematostella* – the oral/midbody boundary and the midbody/aboral boundary. We showed that the oral/midbody boundary is established by the module of four transcription factors: Brachyury, Lmx, FoxA and FoxB. The midbody/aboral boundary is created due to the activity of the transcription factor Sp6-9 (Lebedeva et al., 2021). The whole regulatory principle and the genes involved in the OA patterning of the *Nematostella* embryo showed striking resemblance to the logic and the components of the PA patterning in deuterostome Bilateria (Darras et al., 2018, 2011; Kiecker and Niehrs, 2001; Lebedeva et al., 2021; Nordström et al., 2002; Range, 2018; Range et al., 2013). In contrast to endoderm specification, axial patterning is strongly affected by the knockdowns of LRP5/6 or combined knockdowns of Fz receptors, which demonstrate partial functional redundancy. The fact that LRP5/6 phenotype is phenocopied only by the simultaneous knockdown of all four Fz receptors suggests that all of them are involved in β-catenin signalling. The similarity of the combined *Wnt3*+*Wnt4* KD phenotype, as well as of the combined *Wnt1*+*Wnt4* KD phenotype, to the *LRP5/6* KD and the quadruple Fz gene KD indicates that these three orally expressed Wnt ligands play the main role in the Fz/LRP5/6-mediated OA patterning during early *Nematostella* development. KD phenotype similarity also suggests that, among these three Wnt ligands, Wnt4 appears to be the one predominantly signalling via the aborally expressed Fz5.

Taken together, our data suggest the crucial role of the Wnt/LRP5/6/Fz-mediated signalling in the OA patterning of the sea anemone *Nematostella vectensis*, in which different Fz receptors play partially redundant roles. In contrast, we do not find evidence for the involvement of Fz/LRP5/6-mediated signalling in the specification of the pre-endodermal plate. With this work, we lay the foundation for the future research, which will show whether Fz functions become more distinct at later developmental stages, identify the possible signalling preferences of the different Wnt ligands towards different Fz receptors, and address the role of the non-canonical Wnt pathways in *Nematostella* development. Ultimately, it will be important to understand not only the difference between the functions of the different Fz molecules but also the role of their redundancy and the selective pressures maintaining what appears to be an ancestral Fz redundancy conserved in Cnidaria and Bilateria.

## MATERIALS AND METHODS

### Animals, microinjection and electroporation

Adult *Nematostella vectensis* polyps were kept separated by sex in 16‰ artificial sea water (*Nematostella* medium=NM) at 18°C in the dark. Spawning was induced by placing the polyps into an illuminated incubator set to 25°C for 10 h. The eggs were de-jellied with 3% L-cystein/NM as described previously (Genikhovich and Technau, 2009). Microinjection of the shRNAs and morpholinos and electroporation of shRNAs against maternally expressed transcripts was performed prior to fertilization. For zygotic transcripts, electroporation and microinjection was performed after fertilization. The embryos were raised at 21°C.

### Gene knockdown, mRNA overexpression and inhibitor treatments

shRNA-mediated gene knockdown was performed as described previously (Karabulut et al., 2019). Two independent, non-overlapping shRNAs were used for each gene to make sure that the KD result was specific. Regardless of whether one or more genes was being knocked down, the concentration of the shRNA against each transcript was 500 ng/µl. shRNA against *mOrange* was used as a control (shControl). In cases of simultaneous knockdowns, shControl was used at a concentration corresponding to the maximum combined shRNA concentration used against the genes of interest, i.e. in case of a quadruple knockdown, we used 2000 ng/µl shControl. RNAi efficiency was tested by *in situ* hybridization and quantitative PCR (Fig. S2A-C). For morpholino KDs, all MOs were used at a concentration of 250 µM. The activity of the morpholinos was confirmed by co-injecting each of them with 20 ng/µl *mCherry* mRNA containing the recognition sequence for the respective morpholino oligonucleotide and testing whether mCherry translation was suppressed in comparison with the situation, when the same mRNA was co-injected with a control MO (Fig. S2D), which we have tested previously (Kraus et al., 2016; Lebedeva et al., 2021). To generate the *dnFz1-mCherry* construct, the fragment of *Fz1* cDNA encoding the C-terminal domain (27 amino acids of the protein following the seventh transmembrane domain) was replaced with mCherry-coding sequence. *In vitro* transcribed *dnFz1-mCherry* mRNA was microinjected at a concentration of 250 ng/µl. Control *mCherry* mRNA was injected at a concentration of 75 ng/µl since *mCherry* is ∼3.2 times shorter than *dnFz1-mCherry*. mRNA was synthesized with mMessage mMachine kit (Life Technologies) and purified with the Monarch RNA clean-up kit (NEB). 5 µM 1-azakenpaullone (Sigma) used for the treatments was prepared by diluting 5 mM AZK dissolved in DMSO with NM. An equal volume of DMSO was used to treat the control embryos. The duration of the treatment is described on Fig. 4A. The recognition sequences for the shRNAs, as well as the morpholino sequences are shown in Tables S1 and S2. Accession numbers for the genes used in the study are presented in Table S3.

### *In situ* hybridization and phalloidin staining

*In situ* hybridization was performed as described previously (Kraus et al., 2016) with a single change: the embryos were fixed for 1 h at room temperature in 4%PFA/PBS, washed several times in PTw (1× PBS and 0.1% Tween 20), then in 100% methanol and finally stored in 100% methanol at −20°C. Digoxigenin-labelled RNA probes were detected with anti-digoxigenin-AP Fab fragments (Roche) diluted 1:4000 in 0.5% blocking reagent (Roche) in 1× MAB. After unbound antibody was removed by a series of ten PTw washes of 10 min each, the embryos were stained with a mixture of NBT/BCIP, embedded in 86% glycerol and imaged using a Nikon 80i compound microscope equipped with the Nikon DS-Fi1 camera. For phalloidin staining, the embryos were fixed in 4%PFA/PTwTx (1× PBS, 0.1% Tween 20 and 0.2% Triton X-100) for 1 h at room temperature, washed five times with PTwTx, incubated in 100% acetone pre-cooled to −20°C for 7 min on ice and washed three more times with PTwTx. 2 µl of phalloidin-AlexaFluor488 (ThermoFisher) was added per 100 µl PTwTx, and the embryos were stained overnight at 4°C. After eight 10-min washes with PTwTx, the embryos were gradually embedded in Vectashield (Vector labs) and imaged with the Leica SP8 CLSM.

## Supplementary Material

10.1242/develop.200785_sup1 Supplementary information
