# Supplementary material for: Sea anemone Frizzled receptors play partially redundant roles in oral-aboral axis patterning
Source: Development. 2022 Oct 12;149(19):dev200785. doi: 10.1242/dev.200785 (PMC9720753; doi:10.1242/dev.200785)
Supplement: Supplementary information [file develop-149-200785-s1.pdf]

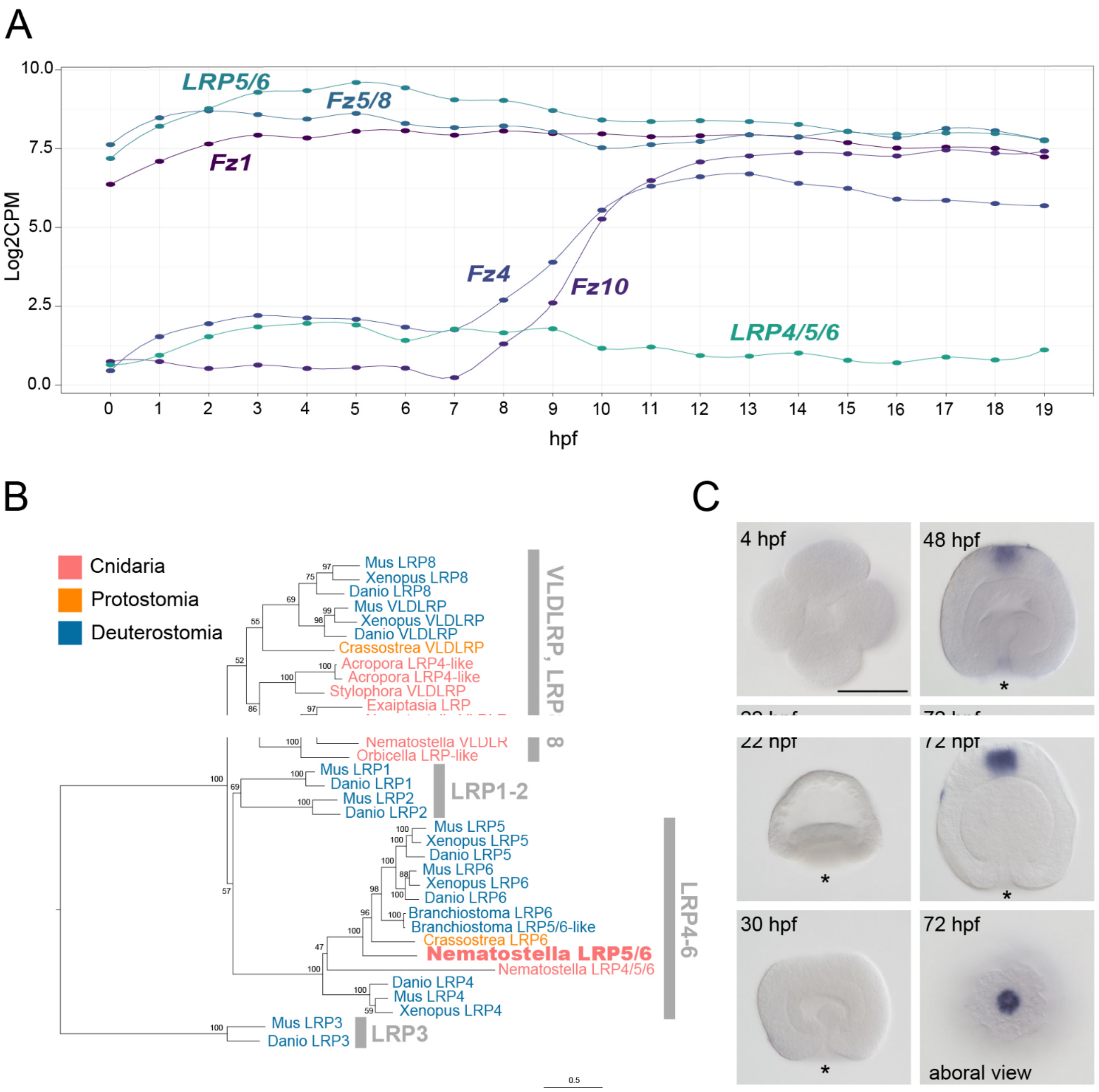

Fig. S1. (A) Expression dynamics of the *LRP5/6*, *LRP4/5/6-like* and *Fz* genes in the first 19 hours of *Nematostella* development according to the NvERTx database (Helm et al., 2013; Warner et al., 2018). (B) Maximum likelihood phylogeny of the LRP proteins (WAG+G4, bootstrap 100). (C) LRP4/5/6-like is expressed in the apical organ of the planula. Scale bar 100 µm.

Development • Supplementary information

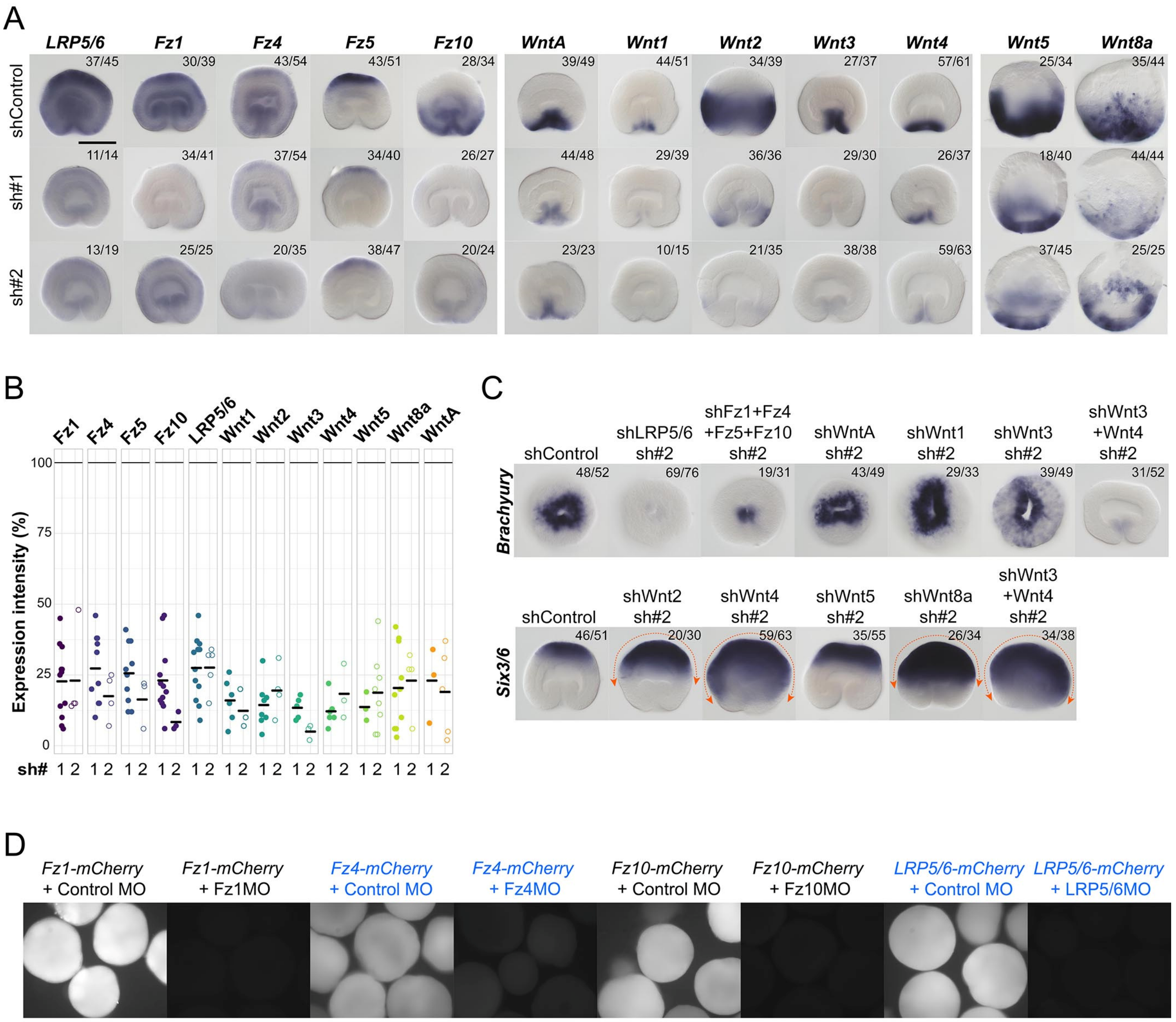

Fig. S2. Controls of the efficiency of the shRNAs and morpholino oligonucleotides. (A) Efficiency of the first (sh#1) and the second (sh#2) shRNA for each gene analyzed by in situ hybridization. *Wnt5* and *Wnt8a* expression has been assessed at mid-blastula stage because unlike all the other genes shown on the figure, *Wnt5* and *Wnt8a* are nearly not expressed at late gastrula stage. All other transcripts are stained in 30 hpf gastrulae. (B) qPCR analysis of the knockdown efficiency of the sh#1 and sh#2 for each gene in relation to shControl (100% expression) at 30 hpf. (C) The phenotypes obtained with the sh#1 (shown on all other figures) are reproduced with minimal differences using the sh#2 in 30 hpf embryos. Oral view is shown for *Bra*, lateral view is shown for *Six3/6*. On (A) and (C), the numbers in the top right corner show the fraction of the embryo demonstrating this phenotype. Scale bar 100 µm.

(D) In vivo fluorescence shows that *mCherry* mRNA carrying the morpholino recognition sequence for the Fz1MO, Fz4MO, Fz10MO or LRP5/6MO is efficiently translated when co-injected into zygotes together with the control morpholino, but not when co-injected with the morpholinos against Fz1, Fz4, Fz10 or LRP5/6MO, respectively.

Development • Supplementary information

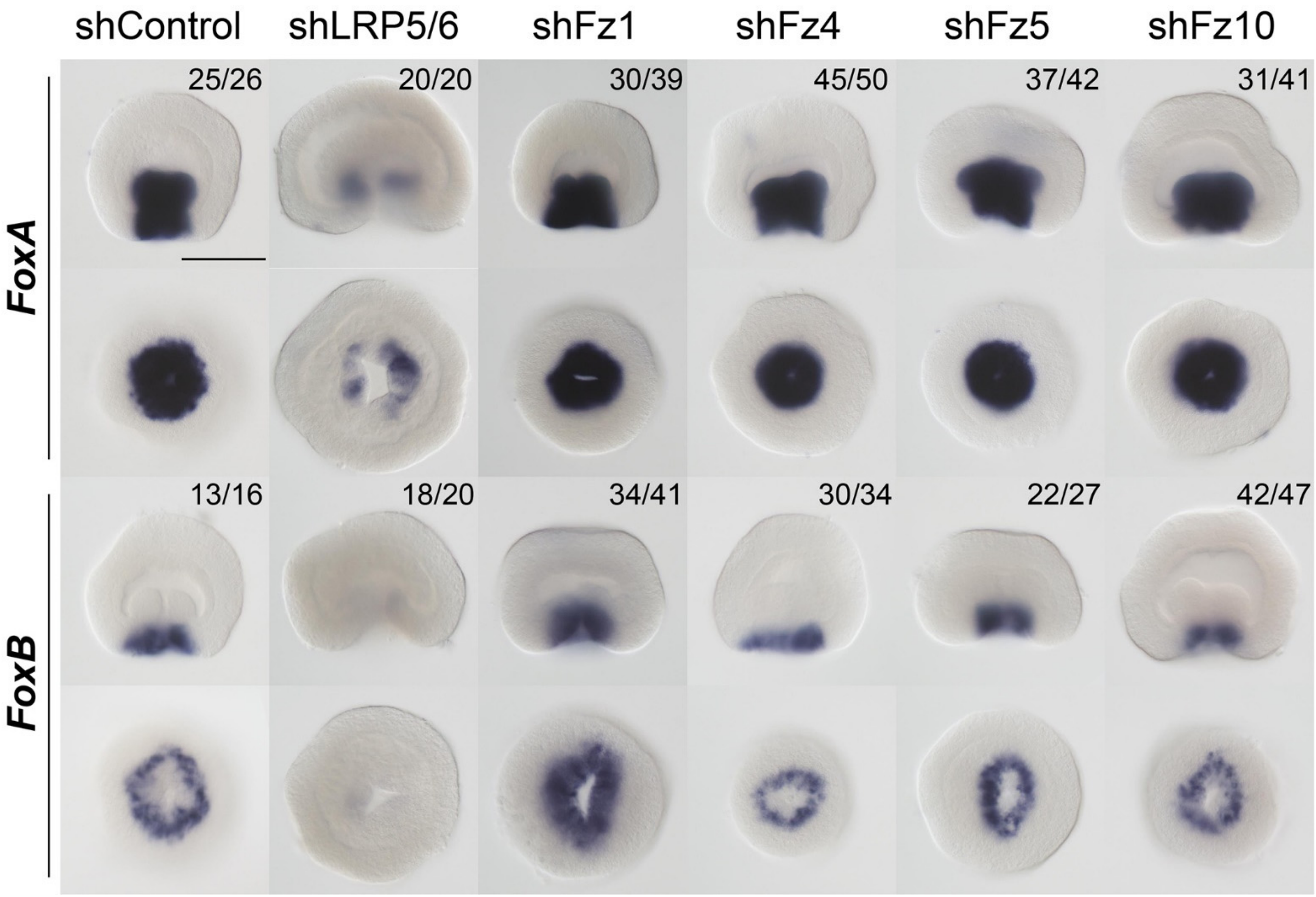

Fig. S3. Expression of the oral markers *FoxA* and *FoxB* upon KDs of *LRP5/6* and individual *Fz* in the 30 hpf gastrula. The numbers in the top right corner show the fraction of the embryo demonstrating this phenotype. For each gene, lateral views (oral end down) on the top, oral views on the bottom. Scale bar 100 µm.

Development • Supplementary information

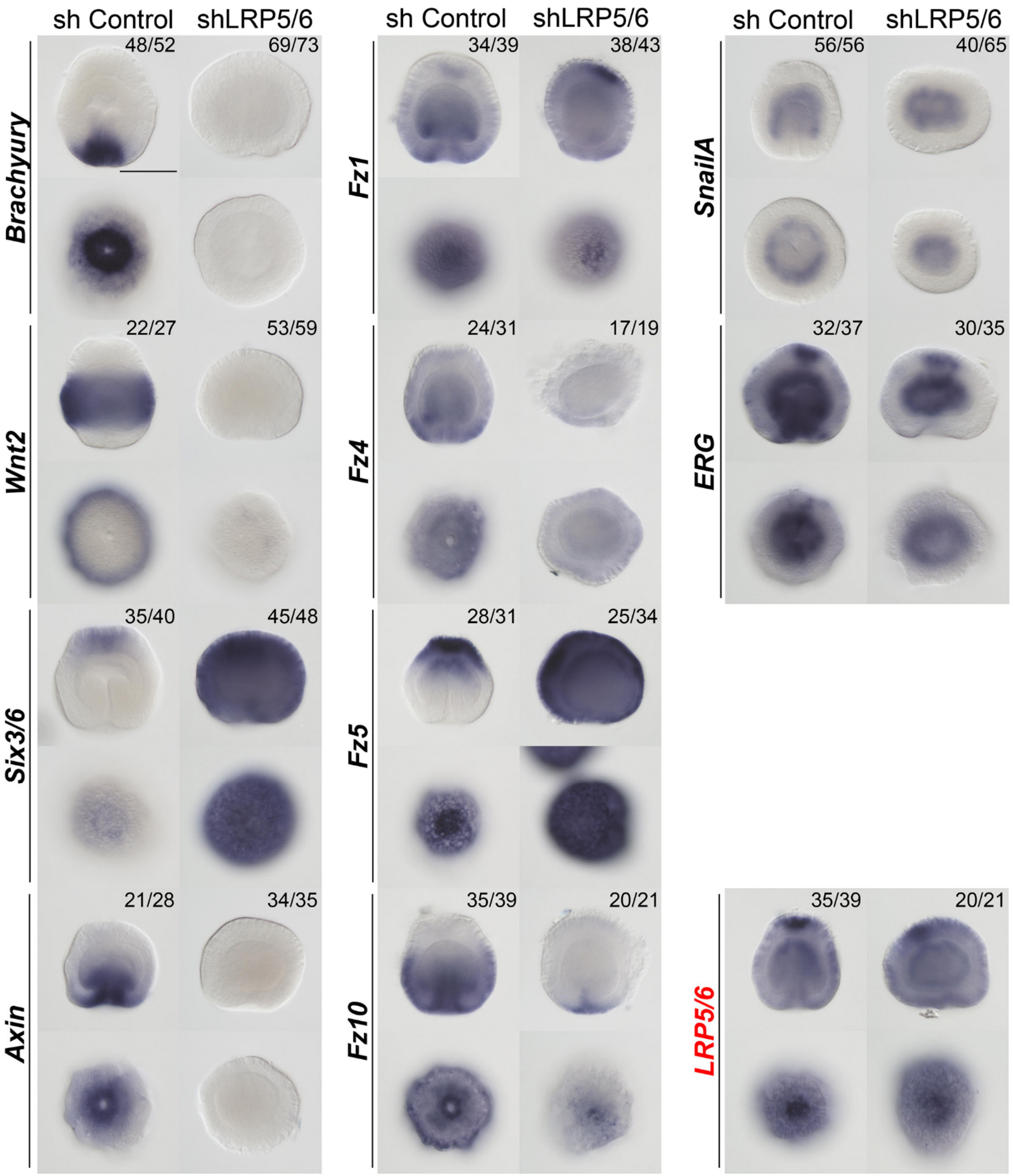

Fig. S4. Marker gene expression in 3 dpf planulae upon *LRP5/6* RNAi. The numbers in the top right corner show the fraction of the embryo demonstrating this phenotype. Scale bar 100 µm. For each gene, lateral views (oral end down) on the top, oral views on the bottom.

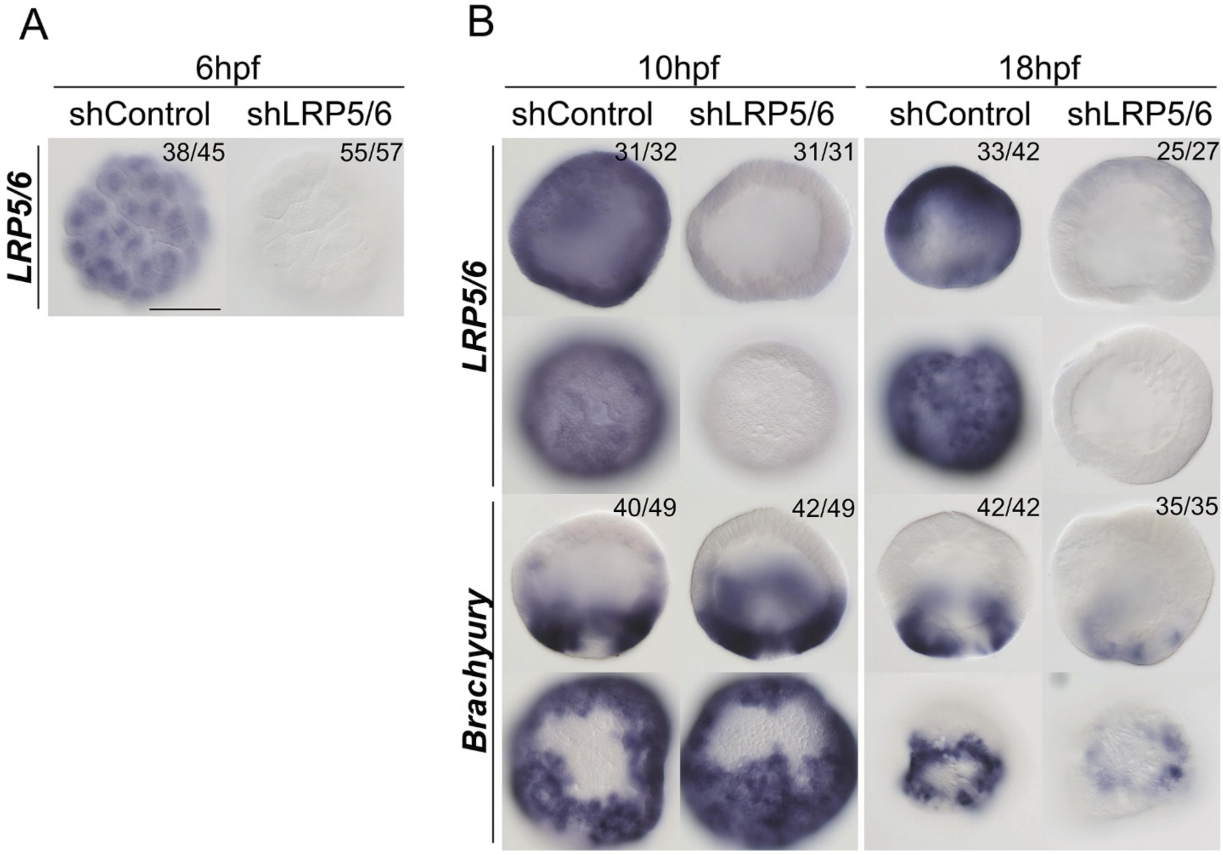

Fig. S5. The onset of the effect of the *LRP5/6* RNAi. (A) *LRP5/6* expression is abolished at 6 hpf. (B) *Bra* expression is not affected at 10 hpf, but starts to be suppressed by 18 hpf. The numbers in the top right corner show the fraction of the embryo demonstrating this phenotype. Scale bar 100 µm. For each gene, lateral views (oral end down) on the top, oral views on the bottom.

Development • Supplementary information

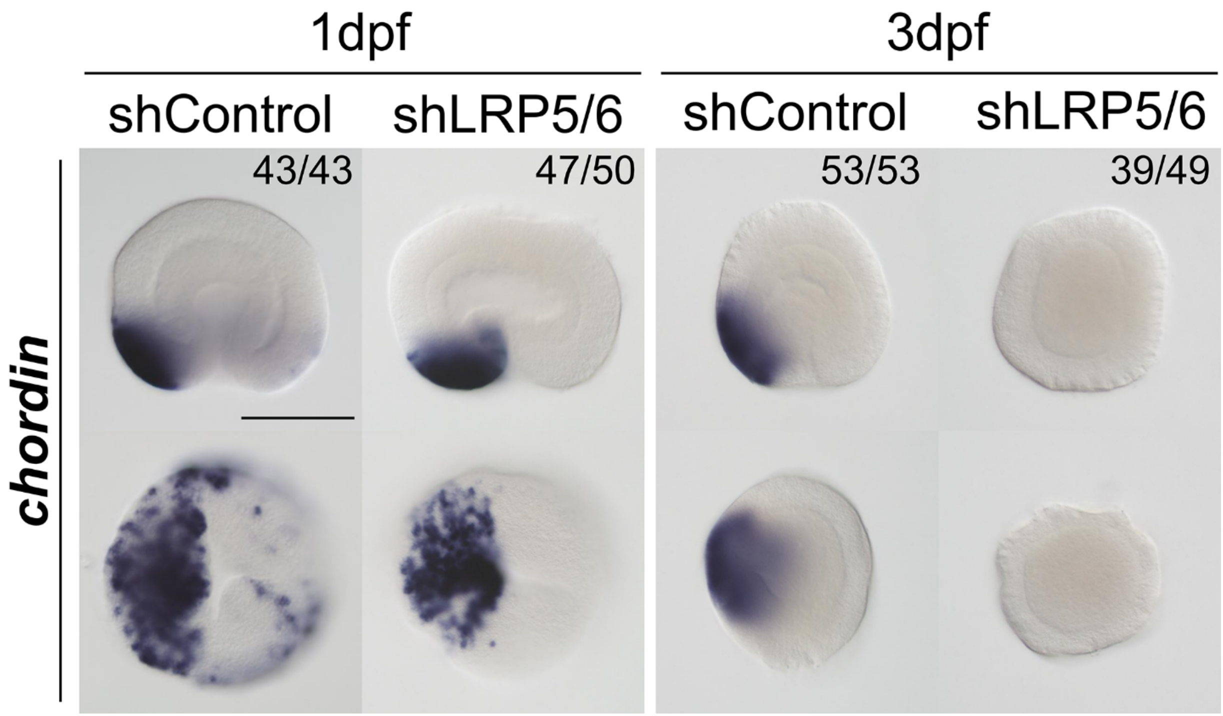

Fig. S6. At late gastrula (1 dpf), asymmetric *Chordin* expression indicates the establishment of the directive axis, which disappears by mid-planula (3 dpf). The numbers in the top right corner show the fraction of the embryo demonstrating this phenotype. Scale bar 100 µm. Lateral views (oral end down) on the top, oral views on the bottom.

Development • Supplementary information

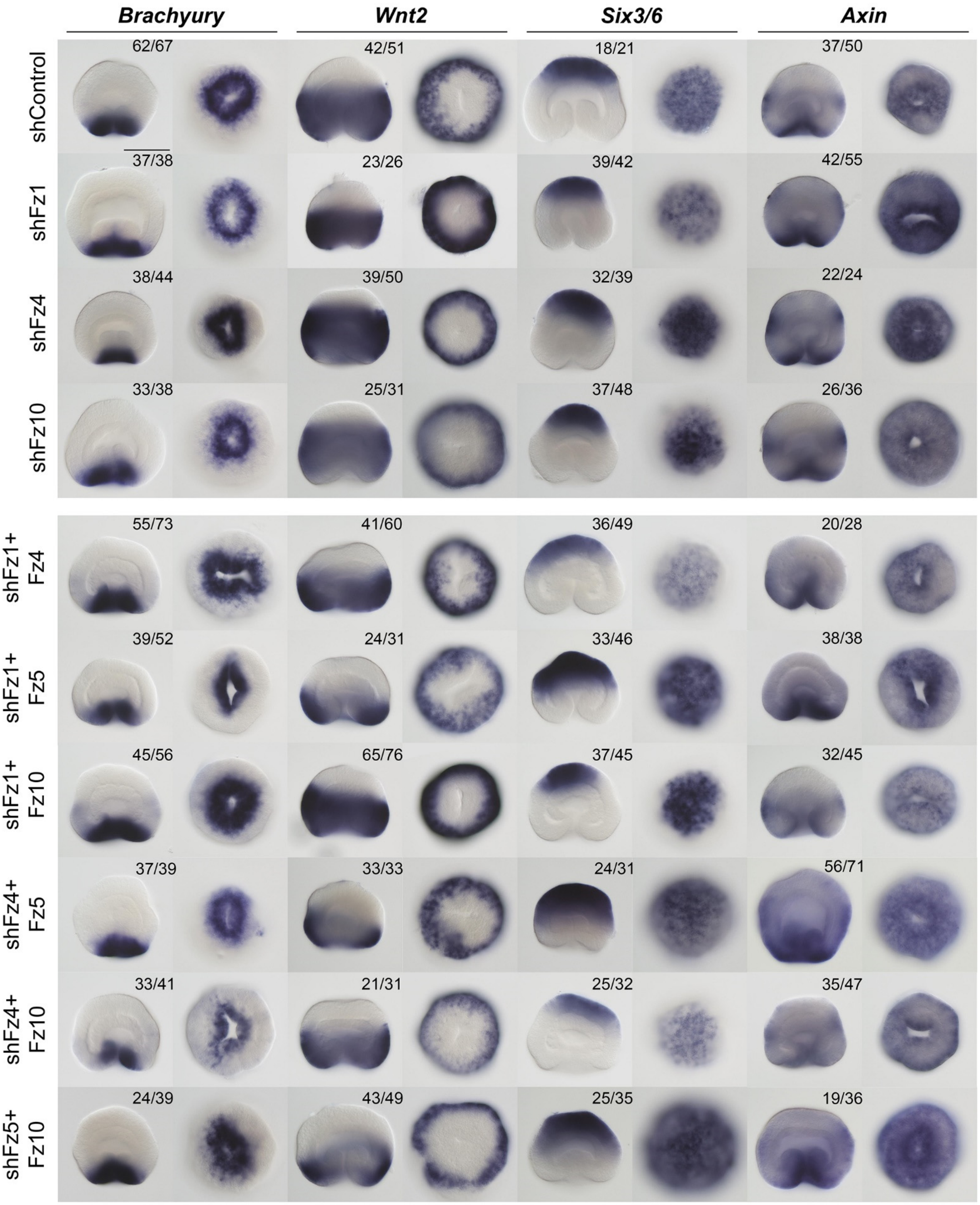

Development • Supplementary information

Fig. S7. Effects of the individual RNAi of the orally expressed Fz genes and effects of the simultaneous RNAi of all possible combinations of two Fz genes on the expression of the β-catenin-dependent markers of different axial domains in the 30 hpf gastrula. The numbers in the top right corner show the fraction of the embryo demonstrating this phenotype. Scale bar 100 µm. For each gene, lateral views (oral end down) on the left, oral (aboral in case of *Six3/6*) views on the right.

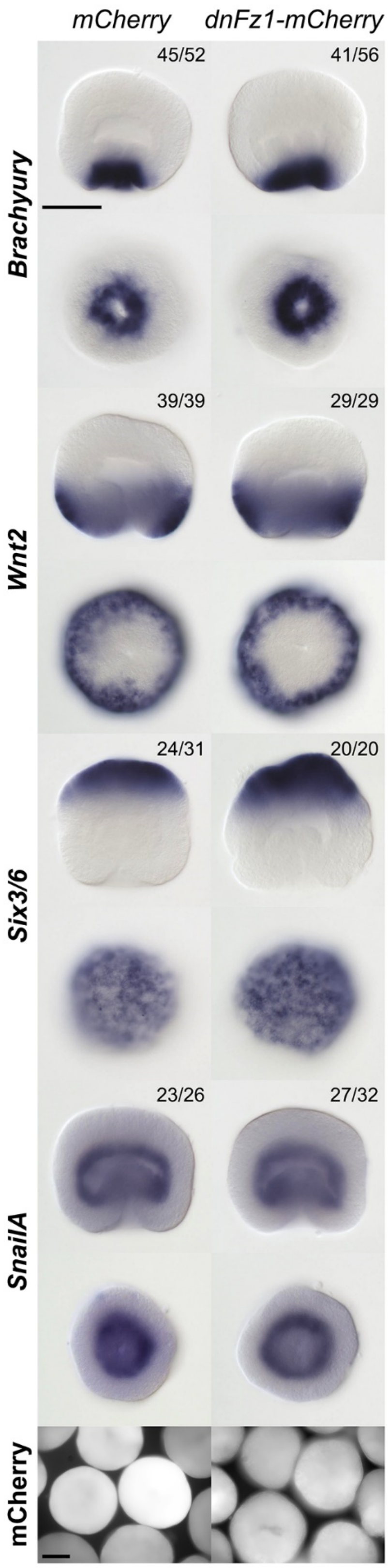

Fig. S8. Microinjection of the *mCherry* mRNA and *dnFz1-mCherry* mRNA show no effect on the expression of the markers of the distinct axial domains in the ectoderm, and on the endodermal marker *SnailA* in the 30 hpf gastrula. The numbers in the top right corner show the fraction of the embryo demonstrating this phenotype. For each gene, lateral views (oral end down) on the top, oral (aboral in case of *Six3/6*) views on the bottom. Lower panel – mCherry fluorescence of the microinjected embryos at 24 hpf. Scale bars 100 µm.

Development • Supplementary information

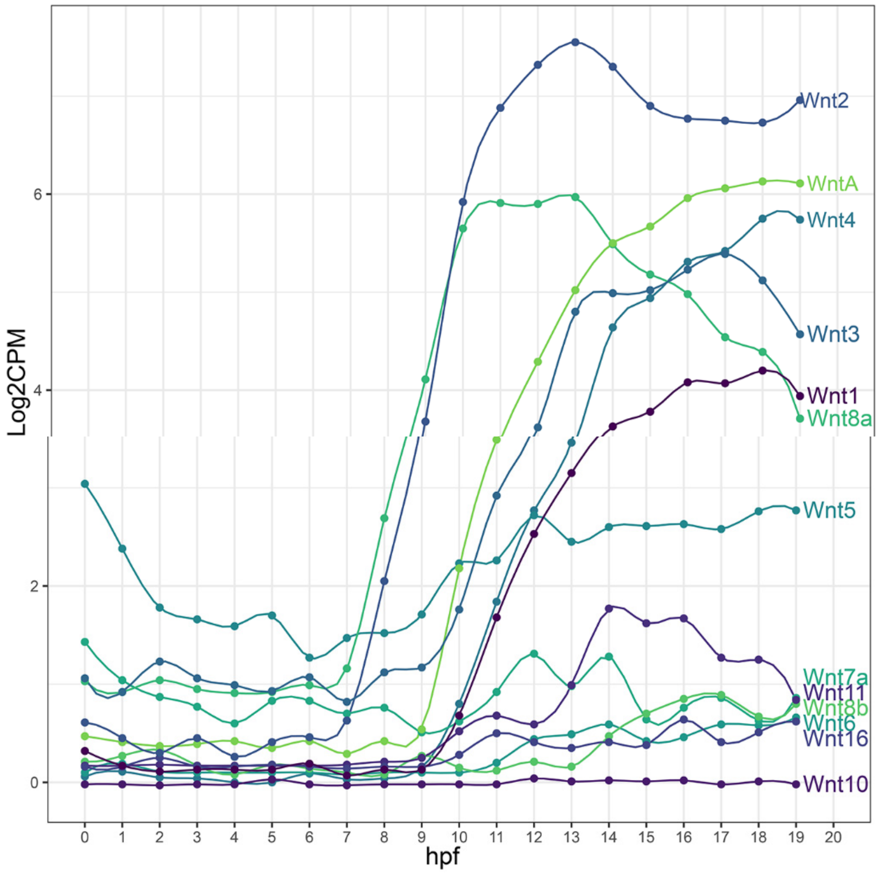

Fig. S9. The dynamics of the *Wnt* gene expression in the first 19 hours of *Nematostella* development according to the NvERTx database (Helm et al., 2013; Warner et al., 2018).

Development • Supplementary information

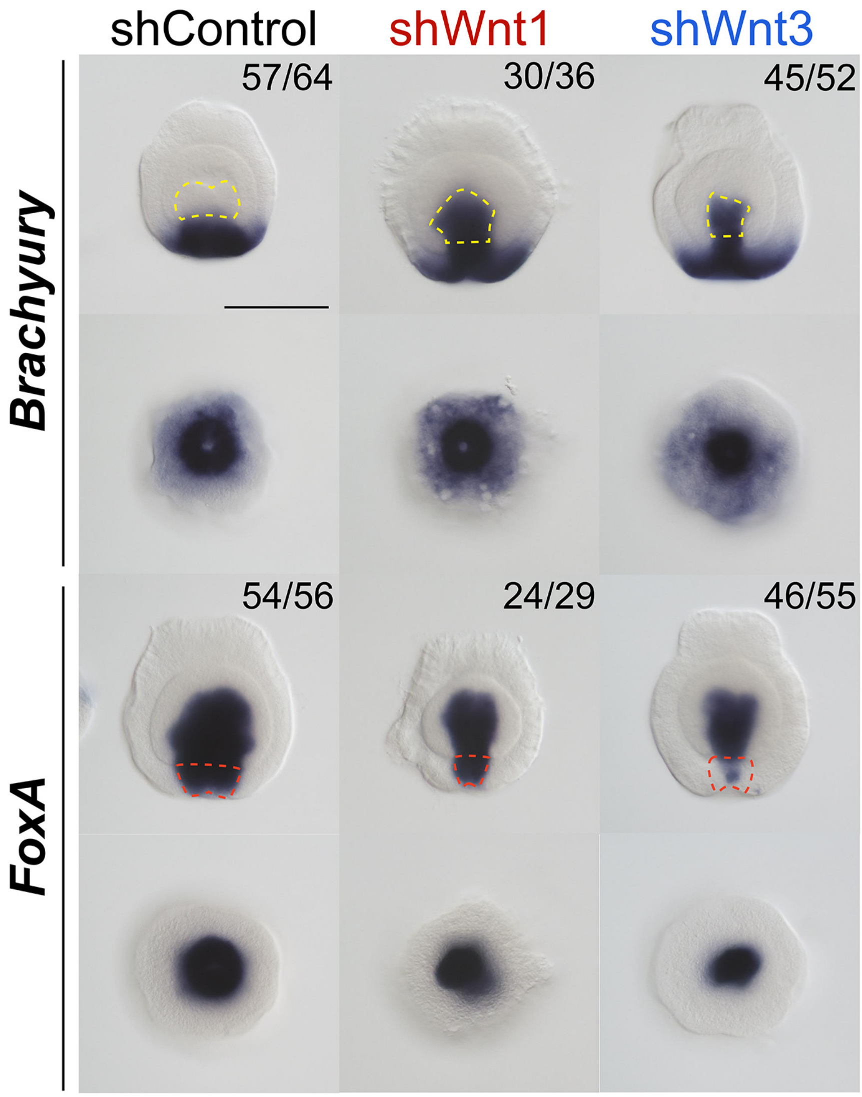

Fig. S10. Analysis of the oral marker expression in the 3 dpf planulae. Similar to the effect seen at the 30 hpf, *Wnt1* and *Wnt3* RNAi result in the expansion of the *Bra* expression to the bottom of the pharynx (yellow dashed line). A similar phenotype was seen in 30 hpf gastrulae upon *FoxB* RNAi (Lebedeva et al., 2021). *Wnt3* RNAi, but not *Wnt1* RNAi results in the suppression of the *FoxA* expression in the outer pharynx (red dashed line). A similar phenotype was seen in 30 hpf gastrulae upon *Bra*, *FoxB*, and *Bra*+*FoxB* RNAi (Lebedeva et al., 2021). For each gene, lateral views (oral end down) on the top, oral views on the bottom. The numbers in the top right corner show the fraction of the embryo demonstrating this phenotype. Scale bar 100 µm.

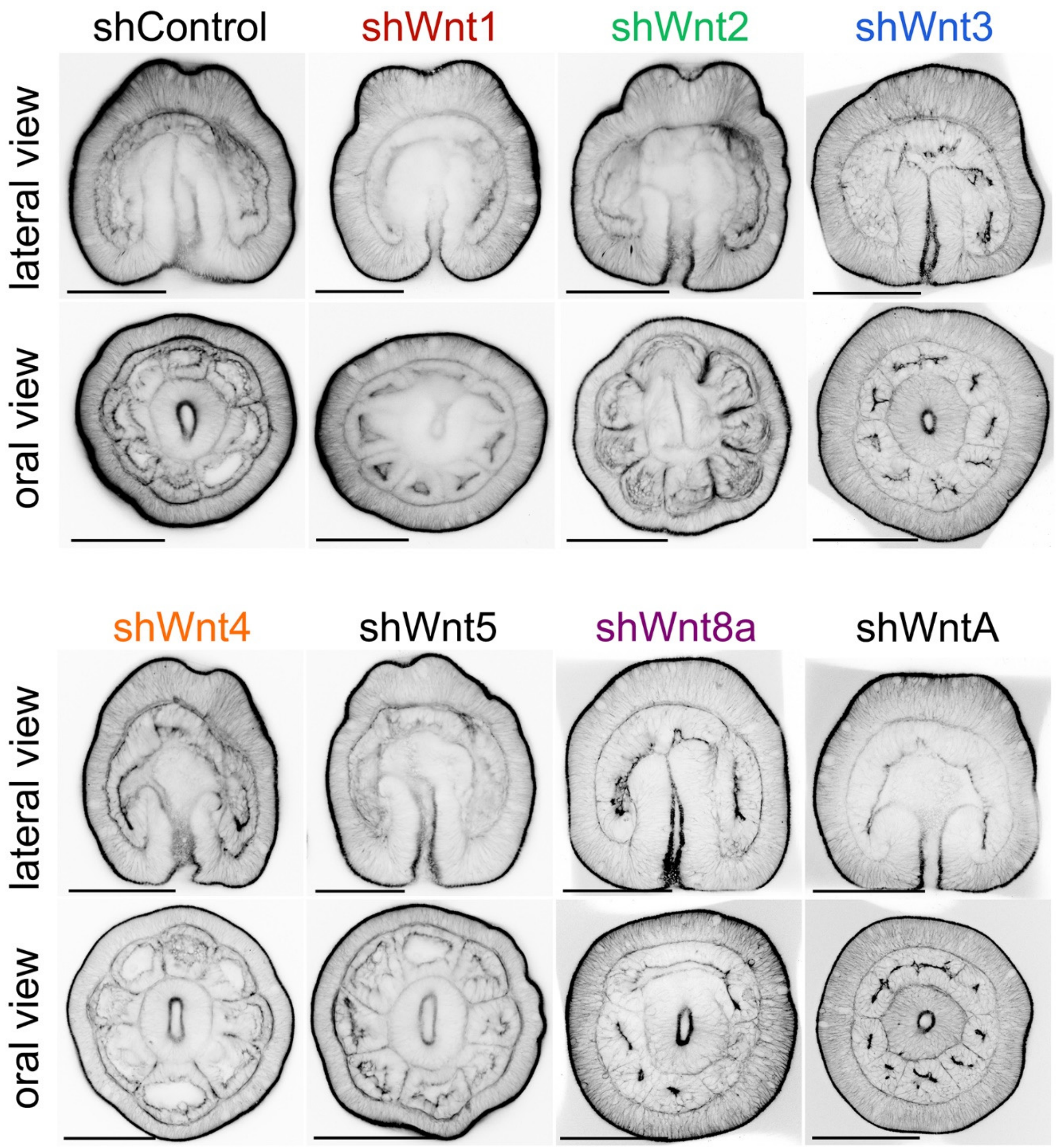

Fig. S11. Phalloidin staining of the 4 dpf planulae upon individual Wnt RNAi. Scale bar 100 μm. shWnt8a and shWntA embryos appear slightly delayed (the last pair of mesenteries has not yet formed), but their general morphology is entirely normal.

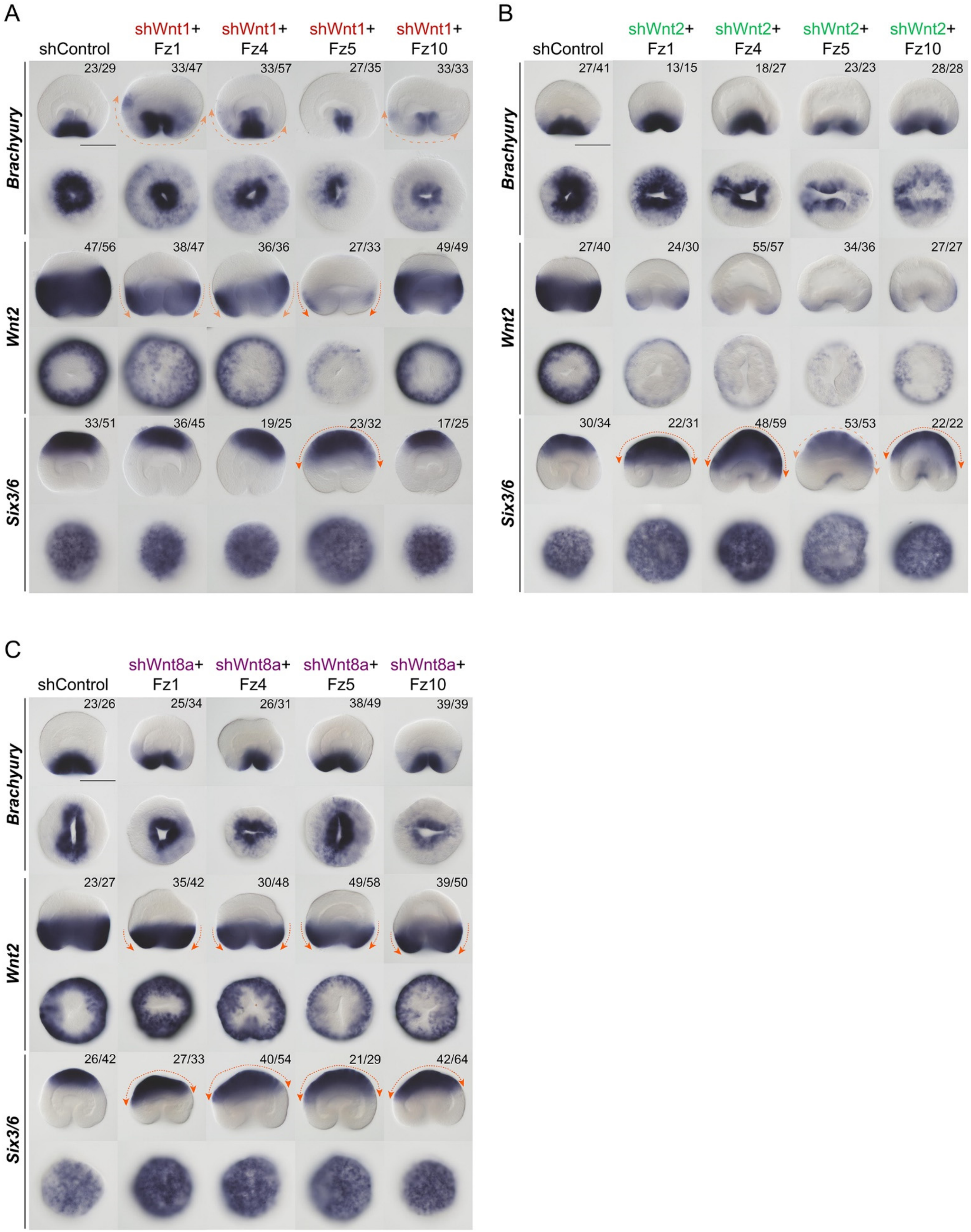

A
shControl
shWnt1+ Fz1
shWnt1+ Fz4
shWnt1+ Fz5
shWnt1+ Fz10
Brachyury
23/29
33/47
33/57
27/35
33/33
Wnt2
47/56
38/47
36/36
27/33
49/49
Six3/6
33/51
36/45
19/25
23/32
17/25
B
shControl
shWnt2+ Fz1
shWnt2+ Fz4
shWnt2+ Fz5
shWnt2+ Fz10
Brachyury
27/41
13/15
18/27
23/23
28/28
Wnt2
27/40
24/30
55/57
34/36
27/27
Six3/6
30/34
22/31
48/59
53/53
22/22
C
shControl
shWnt8a+ Fz1
shWnt8a+ Fz4
shWnt8a+ Fz5
shWnt8a+ Fz10
Brachyury
23/26
25/34
26/31
38/49
39/39
Wnt2
23/27
35/42
30/48
49/58
39/50
Six3/6
26/42
27/33
40/54
21/29
42/64

Fig. S12. Results of the simultaneous KD of *Wnt1* (A), *Wnt2* (B), and *Wnt8a* (C) with different *Fz* genes on the expression of the oral, midbody, and aboral markers at 30 hpf. For each gene, lateral views (oral end down) on the top, oral (aboral in case of *Six3/6*) views on the bottom. The numbers in the top right corner show the fraction of the embryo demonstrating this phenotype. Scale bars 100 µm. The effect of the combined KD of Wnt1+Fz5 and Wnt1+Fz10 on the expression of *Bra*, *Wnt2* and *Six3/6* is very similar to that of the Wnt3+Fz5 and Wnt3+Fz10 RNAi, however, *Wnt2* expression appears weaker in the Wnt1+Fz5 KD than in the Wnt3/Fz5 KD. Wnt2-Fz and Wnt8a-Fz combinations do not seem to show any noticeable synergistic effects.

Development • Supplementary information

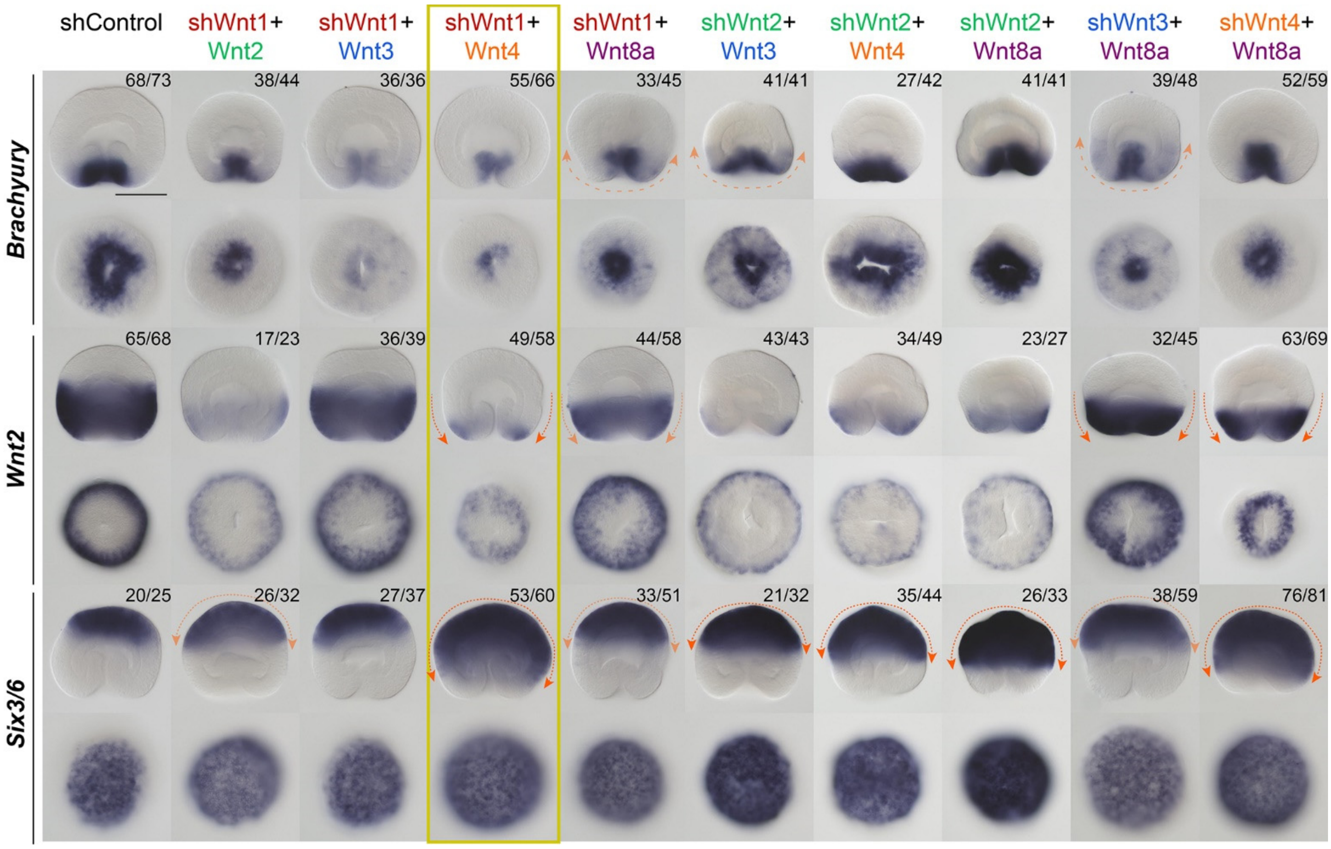

Fig. S13. Results of the double KD of all *Wnt* genes producing and oral-aboral phenotype at 30 hpf upon individual KDs (see Fig. 6A in the main text). Expression of the same oral, midbody, and aboral markers at 30 hpf is shown. For each gene, lateral views (oral end down) on the top, oral (aboral in case of *Six3/6*) views on the bottom. The numbers in the top right corner show the fraction of the embryo demonstrating this phenotype. Scale bar 100 µm. shWnt1+shWnt4 combination (yellow box) produces a phenotype similar to the double KD of *Wnt3* and *Wnt4* (see Fig. 6C in the main text). The expansion of the aboral marker *Six3/6* upon double KD of *Wnt4* and *Wnt8a* appears more pronounced than upon individual KDs of these two *Wnt* genes. Other shWnt combinations did not seem to elicit a synergistic effect on the expression of *Bra*, *Wnt2*, and *Six3/6* in comparison to their individual KDs.

Development • Supplementary information

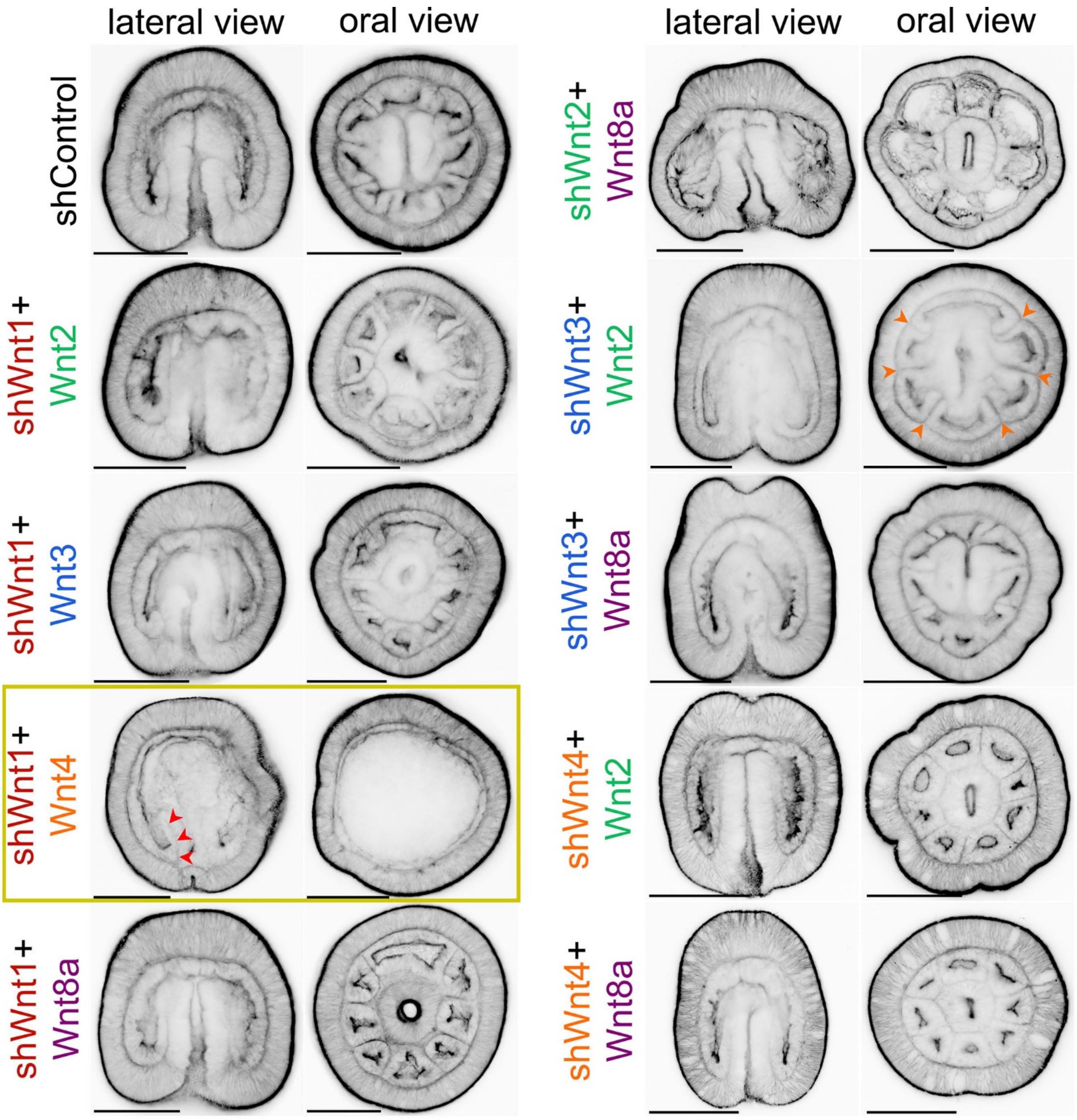

Fig. S14. Phalloidin staining of the 4 dpf planulae upon double KDs of all *Wnt* genes producing and oral-aboral phenotype at 30 hpf. Scale bar 100 µm. Similar to the shWnt3+Wnt4 combination (see Fig. 3D in the main text for comparison), shWnt1+Wnt4 (yellow box) results in a loss of the mouth, pharynx and mesenteries. However, 1-2 residual mesenteries may still be visible (red arrowheads). shWnt3+Wnt2 combination regularly resulted in embryos having 6 rather than 8 regularly spaced mesenteries (orange arrowheads). Other double knockdowns did not lead to any obvious phenotypes at the 4 dpf stage.

Development • Supplementary information

Table S1. Target sequences of the shRNAs

shControl	GCGAGTTCTTCTACAAGGTGA
shLRP5/6 sh#1	GAGAGCCTTCCACTTGTAA
shLRP5/6 sh#2	GAGGAATCGTCGCATCTAT
shFz1 sh#1	GAAGGCTGCACGGTTATTT
shFz1sh#2	GCTTGCAATGAGCCTATCA
shFz4 sh#1	GTTCAAAGCACCGAGTCTT
shFz4sh#2	GCCTGAGAAACCTAGACCA
shFz5 sh#1	GCGGAATAGGCTACAATTT
shFz5 sh#2	GCCGGAATGAAATGGTCAA
shFz10 sh#1	GGATGAACTGACAGGTCTT
shFz10 sh#2	GGACAGTACCAGCAATACA
shWntA sh#1	GGATAACATGGGCAAGACA
shWntA sh#2	GGCGTACTATGCCAAACTT
shWnt1 sh#1	GGAGGATGCAGTGATAACA
shWnt1 sh#2	GGGATTTCCGTGCTCAGAT
shWnt2 sh#1	GAGGGCGTTGATGAACTTA
shWnt2 sh#2	GAGGATTCGCCCAATTACT
shWnt3 sh#1	GGAAGACAGTGCAACTACA
shWnt3 sh#2	GAGACCTCACCAAACTACT
shWnt4 sh#1	GCTTCGCTAGTGTACTCAA
shWnt4 sh#2	GAAATTCGATGGAGCTACT
shWnt5 sh#1	GGTGCCGATGCAAGTTTCA
shWnt5 sh#2	GCTCGGACTCTTATGAACT
shWnt8a sh#1	GGCGCAAAGCTGTTAAGAA
shWnt8a sh#2	GCAGCCTGGTCTTCCTAAA

Table S2. Morpholino sequences

Name	Sequence	Reference
Control MO	GATGTGCCTAGGGTACAACAACAAT	(Kraus et al., 2016; Lebedeva et al., 2021)
Fz1MO	GCATAATCCCGGCGATTAAACTACG	This paper
Fz4MO	GTGACATTTTGCACGAATGGAGAAC	This paper
Fz10MO	AAGCTAAACGCTTAGCCCCCATATC	(Wijesena et al., 2022)
LRP5/6MO	ACAAAACAACTTTGGCGAACATCCT	This paper

Development • Supplementary information

Table S3. Accession numbers

Gene name	Accession	NvERTx	NVE
Wnt4	XP_001623100	NvERTx.4.48250	NVE17746
Wnt5	XP_001630693	NvERTx.4.146133	NVE1780
Wnt8a	XP_001630032	NvERTx.4.106136	NVE2847
WntA	XP_001637670	NvERTx.4.132141	NVE12095
Wnt1	XP_001641494	NvERTx.4.56072	NVE12960
Wnt2	XP_032238966	NvERTx.4.114443	NVE21992
Wnt3	XP_032241388	NvERTx.4.107815	NVE17595
Fzd1	XP_001647540	NvERTx.4.186145	NVE7119
Fzd5	XP_001634995	NvERTx.4.67323	NVE19736
Fzd10	XP_032235151	NvERTx.4.80873	NVE1835
Fzd4	XP_001622965	NvERTx.4.95377	NVE18184
LRP5/6	XP_032222612	NvERTx.4.111182	NVE16348
FoxA	XP_001634555	NvERTx.4.73097	NVE20630
ERG	XP_032236866	NvERTx.4.84016	NVE25536
SnailA	XP_032243077	NvERTx.4.57438	NVE13986
Axin	XP_001640692	NvERTx.4.66791	NVE22529 (no good NVE model)
FoxB	XP_001631625	NvERTx.4.229455	NVE26195
Chordin	XP_001633548	NvERTx.4.87650	NVE22735
Brachyury	XP_032233913	NvERTx.4.100809	NVE770
Six3/6	XP_032228424	NvERTx.4.97387	NVE12346

NVE gene models can be accessed at
https://figshare.com/articles/Nematostella_vectensis_transcriptome_and_gene_models_v2_0/807696
NvERTx transcripts can be accessed at
http://nvertx.ircan.org/ER/ER_plotter/home

References

Helm, R. R., Siebert, S., Tulin, S., Smith, J. and Dunn, C. W. (2013). Characterization of differential transcript abundance through time during Nematostella vectensis development. *BMC genomics* **14**, 266.

Kraus, Y., Aman, A., Technau, U. and Genikhovich, G. (2016). Pre-bilaterian origin of the blastoporal axial organizer. *Nat Commun* **7**, 11694.

Lebedeva, T., Aman, A. J., Graf, T., Niedermoser, I., Zimmermann, B., Kraus, Y., Schatka, M., Demilly, A., Technau, U. and Genikhovich, G. (2021). Cnidarian-bilaterian comparison reveals the ancestral regulatory logic of the β-catenin dependent axial patterning. *Nat Commun* **12**, 4032.

Warner, J. F., Guerlais, V., Amiel, A. R., Johnston, H., Nedoncelle, K. and Rottinger, E. (2018). NvERTx: a gene expression database to compare embryogenesis and regeneration in the sea anemone Nematostella vectensis. *Development (Cambridge, England)* **145**.
Wijesena, N., Sun, H., Kumburegama, S. and Wikramanayake, A. H. (2022). Distinct Frizzled receptors independently mediate endomesoderm specification and primary archenteron invagination during gastrulation in Nematostella. *Developmental biology* **481**, 215-225.
