# Supplementary material for: Sea anemone Frizzled receptors play partially redundant roles in oral-aboral axis patterning
Source: Development. 2022 Oct 12;149(19):dev200785. doi: 10.1242/dev.200785 (PMC9720753; doi:10.1242/dev.200785)
Supplement: Supplementary file 1 [file develop-149-200785_review_history.pdf]

Sea anemone Frizzled receptors play partially redundant roles in oral-aboral axis patterning

Isabell Niedermoser, Tatiana Lebedeva and Grigory Genikhovich
DOI: 10.1242/dev.200785

Editor: Cassandra Extavour

Review timeline
Original submission: 22 March 2022
Editorial decision: 3 May 2022
First revision received: 10 August 2022
Accepted: 12 September 2022

Original submission

First decision letter

MS ID#: DEVELOP/2022/200785

MS TITLE: Sea anemone Frizzled receptors play partially redundant roles in the oral-aboral axis patterning

AUTHORS: Isabell Niedermoser, Tatiana Lebedeva, and Grigory Genikhovich

I have now received all the referees' reports on the above manuscript, and have reached a decision. The referees' comments are appended below, or you can access them online: please go to BenchPress and click on the 'Manuscripts with Decisions' queue in the Author Area.

As you will see, the referees express considerable interest in your work, but have some significant criticisms and recommend a substantial revision of your manuscript before we can consider publication. The most important point, on which multiple reviewers and myself agree, is that some kind of experimental assessment of the efficacy of the knockdown experiments must be included in the MS. If you are able to revise the manuscript along the lines suggested, which may involve further experiments, I will be happy receive a revised version of the manuscript. Your revised paper will be re-reviewed by one or more of the original referees, and acceptance of your manuscript will depend on your addressing satisfactorily the reviewers' major concerns. Please also note that Development will normally permit only one round of major revision. If it would be helpful, you are welcome to contact us to discuss your revision in greater detail. Please send us a point-by-point response indicating your plans for addressing the referee’s comments, and we will look over this and provide further guidance.

Please attend to all of the reviewers' comments and ensure that you clearly highlight all changes made in the revised manuscript. Please avoid using 'Tracked changes' in Word files as these are lost in PDF conversion. I should be grateful if you would also provide a point-by-point response detailing how you have dealt with the points raised by the reviewers in the 'Response to Reviewers' box. If you do not agree with any of their criticisms or suggestions please explain clearly why this is so.

© 2022. Published by The Company of Biologists under the terms of the Creative Commons Attribution License (https://creativecommons.org/licenses/by/4.0/).

Reviewer 1

Advance summary and potential significance to field

This is an interesting, well convinced and well written manuscript, providing solid data on the role of the extracellular components of the Wnt Beta catenin signaling pathway during embryogenesis and larval formation in the sea anemone Nematostella vectensis. By performing a comprehensive set of knockdown (KD) experiments, the authors show the respective role of LRP5/6 and Frizzled receptors in patterning the oral aboral axis. They show that the four Nematostella Frizzled receptors are partially redundant. They also convincingly contradict recently published results (Wijesena et al. 2022) on the function of Frizzled 1 and Frizzled 10 during Nematostella embryogenesis. Finally, they address the role of the Wnt ligands in oral aboral patterning and show that double KD Wnt3/Wnt4 is sufficient to phenocopy LRP5/6 or Frizzleds KD. This study is an excellent addition to the literature as it represents the most comprehensive analysis of the role of the Wnt pathway extracellular components performed in a cnidarian.

Comments for the author

I would suggest a few experiments to strengthen the data and some corrections in the text and figures.

Major comments:
KD efficiency following shRNA injection should be estimated by qPCR. This is particularly relevant for the KD of the Wnt genes as these have not been confirmed by morpholino injection and many Wnt shRNA injections lead to no clear phenotype.
It is reported on page 14 that Wnt1 and Wnt3 KD “resulted in a reduction of the expression of the oral marker Bra, and its expansion to the bottom of the pharynx”. I however cannot see this in the images provided in Figure 6. The authors would need to show more convincing data and/or downplay this result.
The rational for focusing on the role of Wnt3 and Wnt4 for multiple knockdown experiments (“KDs of Wnt3 and Wnt4 resulted in the strongest patterning changes”) is questionable as Wnt3 KD phenotypes are barely noticeable. The authors conclude from the reported Wnt3/Wnt4 KDs that “these two orally expressed Wnt ligands play the main role in the Fz/LRP5/6-mediated OA patterning during early Nematostella development. KD phenotype similarity also suggests that among these two Wnt ligands, Wnt4 appears to be the one predominantly signalling via the aborally expressed Fz5.” (page 17). Without fully addressing the role of the other Wnts, these conclusions seem premature. I would suggest either downplaying the conclusions on the role of Wnt3 and Wnt4 in Fz/LRP5/6-mediated OA patterning or fully investigating the role of the other Wnts (most importantly Wnt2 for which KD results in clear oral expansion of Six3/6) performing double KD experiments with the other Wnts and Frizzleds, as they did for Wnt3 and Wnt4.

Minor comments:
Figure 1. How was the picture “Fz10 10hpf” oriented? The staining on the lower part of embryo appears weaker than on the upper part. Considering that Fz10 expression at gastrula stage is restricted to the oral side, shouldn’t we reverse the “Fz10 10hpf” image?
Page 6. In situ hybridizations of Fz1, Fz5 and Fz10 at several developmental stages have been already published in Leclère et al. 2016 and Wijesena et al. 2022. This prior work should be acknowledged when describing Figure 1 in the first section of the results.
Page 5. “At the onset of gastrulation, Fz1, Fz4 and LRP5/6 are weakly expressed ubiquitously.” As in situ hybridization is not a quantitative method, this sentence is misleading. Moreoever, according to Supp Figure 1, those three genes do not seem to be particularly lowly expressed at this stage. The word ‘weakly’ should be deleted.
Page 5. “aboral ectoderm appears to be free of Fz1 mRNA.” This cannot be assessed by in situ hybridization. Please rephrase.
Figure 2. In the triple injection shFz4+5+10, Bra and Wnt2 seem to be expressed on only one side of the blastopore. Is this systematically observed? If not, this should be specified in the figure legend; if it is, this information must be mentioned in the results. Could this be linked to the peculiar morphology of these injected larvae?
Page 9 “manifest itself after the RNAi.” A word is missing: “RNAi injection”?

© 2022. Published by The Company of Biologists under the terms of the Creative Commons Attribution License (https://creativecommons.org/licenses/by/4.0/).

Page 11. “Individual Fz RNAi did not lead to significant morphological defects apart from a slight gastrulation delay in Fz1 and Fz10 RNAi, and a previously reported slight shortening of the OA axis in Fz5 RNAi (Leclère et al., 2016).” Please provide more information about these delays. Also, were apical organs reduced in Fz5 shRNA-injected larvae as described for Fz5 MO?
Page 14. “RNAi of Wnt5, which was not very efficient with both shRNAs we used)”. A parenthesis is missing.
Supplementary Table 2. Please indicate whether the MO sequences used in this manuscript were new or already published. Notably, is Fz10 MO the same as the recently published one (Wijesena et al. 2022)?
Please indicate in the Material and Methods and/or in the supplementary material the quantity/concentration used for each shRNA, Morpholino and mRNA injections (single, double, triple and quadruple).
Supp Figure 5. Correct AXIN by Axin.
Supp Figure 8. Correct dnFz-mCherry with dnFz1-mCherry in the figure legend.

Reviewer 2

Advance summary and potential significance to field

Niedermoser et al. provide a neat demonstration of how the canonical Wnt pathway patterns the oral-aboral axis in the cnidarian Nematostella vectensis. The study further shows that cWnt pathway additionally modulates the directive axis, and that endoderm specification is likely dependent on maternal beta-catenin signal. Combinatorial loss-of-function experiments show that the receptor LRP5/6 plays a major role, that the different Frizzled receptors have partially redundant roles, and that the ligands Wnt3 and Wnt4 appear to be particularly relevant for the set up of the oral-aboral axis and the development of endodermal structures.
The study is well done, and the images provided are of high quality. Eventual discrepancies with previous data and among different approaches are convincingly addressed.
This study represents a solid and durable contribution to our understanding of body patterning in a relevant cnidarian model.

Comments for the author

I have one major recommendation, and some further minor comments (in order of appearance).

M1. Given that the phenotypes observed are not independent of the knockdown approach used (shRNA, MOs, dominant negative constructs) and that in some cases no effect was observed, the Authors need to:
- provide further details on the knockdown experiments, in particular regarding the concentrations of shRNA and MO and which controls where used (for example, it appears that a generic control MO was used for all experiments, instead of a gene-specific one; no information is provided on the RNAi control).
- asses independently the efficiency of KDs, as in situ hybridization alone is not quantitative, and stainings can be misleading. This is best exemplified by the case of Wnt2 KD: in supplementary figure 2A we see that the two shRNAs against Wnt2 suppress the expression of Wnt2 itself (with the second shRNA against Wnt2 having a stronger effect), however the same KD would be judged as less efficient when considering only figure 6A. This would allow to further clarify the unsuccessful KD of Wnt5 (judged "not very efficient", page 14) and WntA. I recommend performing a stage-specific quantification of mRNA levels, with qPCR, as already done by the same Authors in Lebedeva et al., 2021.

m1: This is largely a matter of personal taste, but I would recommend starting the Introduction section with the explanation of the roles and the evolution of Wnt signaling, and introduce only later the details of the pathway (which non-experts might find harder to follow).
m2. The sentence "a typical beta-catenin loss-of-function phenotype" (page 3) needs a reference.
m3. Please move the technical details (e.g. "from cDNA") at the beginning of Results section to the Methods.
m4. The fact that LRP4/5/6-like displays an anomalous intracellular domain does not necessarily rule out all involvement in cWnt signaling: it could still modulate the signal, for example acting as

© 2022. Published by The Company of Biologists under the terms of the Creative Commons Attribution License (https://creativecommons.org/licenses/by/4.0/).

a dominant negative. Instead, as it is expressed only later in the apical organ region, we can conclude that it does not take part in patterning the OA axis. Please, rephrase.
m5. The multiple KD of Fz4, Fz5 and Fz10 shows a defect in the radial expression of Bra and Wnt2 (Figure 2) and the same combinatorial KD at planula stage show a defect in the organization of the mesenteries (Figure 3C). Additionally, MO against Fz10 alone caused a defect in the organization of the mesenteries (Figure 5B) - currently interpreted as a delay (pg.11). These findings could instead point to a role of (some) Fz receptors in setting up the directive axis, together with LRP5/6 (Supplementary Figure 6). Please, comment these data.
m6. The rationale for focusing on Wnt3 and Wnt4 KDs as they present the "strongest patterning changes" (pg. 14) is, in my opinion, not justified: the changes in patterning for Wnt8a and Wnt2 KDs are also strong, only that they affect the aboral/midbody boundary. Additionally, also Wnt1 KD shows an interesting phenotype in the Bra expression domain. Please, rephrase.
m7. Please correct "Fig. 6C" (last line of page 14) to "Fig. 6D".
m8. One of the main conclusions of the manuscript is that Wnt3 and Wnt4 are required for the maintenance of the directive axis. It would be good to provide a phalloidin-stained planula stage, showing the organization of the mesenteries in the case of KD with shWnt3 and shWnt4, separately – in complement of the combinatorial KD.
m9. Some bibliographic references are missing – e.g. when describing the expression dynamics of Fz receptors in Figure 1 and the KD phenotypes (Figure 2). In particular, the paper Lebedeva et al., 2021 is widely cited, but several genes were identified in other studies (eg. Sinigaglia et al., 2013 for Six3/6; Leclere et al., 2016 for Fz5 and Wijesena et al., 2022 for Fz1 and Fz10).
m10. In all figures:
- I recommend changing the current style of labels (for example) "shFz1 +Fz4 +Fz10" to the more specific "shFz1 + shFz4 + shFz10";
- please specify the developmental stage/number of hours post fertilization in figure legends, for both main manuscript and supplementary section.
m11. Figure 1: I am confused by the expression of Fz10 (compare 10 hpf and 15 hpf): is the orientation of the embryo correct?
m12. Figure 1: The removal of Fz1 expression from the endodermal plate is not clear, in particular when comparing the 10 and 15 hpf stages. I recommend providing a clearer image, if possible.

<u>Reviewer 3</u>

Advance summary and potential significance to field

Genikhovich and colleagues present here a functional analysis of the roles for frizzled receptors in control of patterning the primary body axis in Nematostella.
The detailed nature of the Wnt signaling mechanisms controlling axis specification in this organism have not yet been fully resolved, in particular the correspondent roles between the several Wnts and Frizzled receptors across the oral-aboral axis in that organism. The study identifies an lrp5/6 factor expressed dynamically in embryogenesis and similar to Fz5 whose expression gradually restricts aborally.
lrp5/6 inhibition caused enlargement of ectodermal aboral identity (six3/6) at the expense of oral identities (bra, wnt2) but without causing gastrulation failure or endoderm specification.
Knockdown of fzd5 partially phenocopied this effect and inhibition of Fz1+Fz10+Fz4+Fz5 enhanced these phenotypes. The authors then identify Wnt3 and Wnt4 as acting together. Together the study identifies a molecular pathway responsible for the early establishment of the oral/aboral axis.

The use of Wnt signals to control polarity of the primary body axis is likely ancient, indicating the importance of understanding the detailed Wnt axis specification mechanisms will be of broad interest to developmental biologists.
The study is strongly supported and clearly written. I have only a few minor suggestions below.

Comments for the author

Minor comments The authors note some discrepancy between their analysis and another lab’s previously published work regarding Fz1 and Fzd10. However, I find the present study to be compellingly executed and an important addition to the field. In some cases, the discrepancy could

© 2022. Published by The Company of Biologists under the terms of the Creative Commons Attribution License (https://creativecommons.org/licenses/by/4.0/).

also be due to the nature of the perturbations, with dominant negative Fz1 expression potentially able to disrupt other Fz-dependent signaling.
On p.9 it seems possible that a perdurance of LRP5/6 protein, either maternally deposited or already translated in early embryogenesis, could potentially be involved in endoderm specification. The language the authors use is indeed careful (“we do not find evidence of its involvement…”) but a possible explanation of inability to sufficiently reduce any early embryonic LRP5/6 protein should be more fully elaborated in discussion. In general, I think it is difficult to make evidence of absence arguments from morpholino and RNAi experiments, so this issue is also a possible concern with the model that Wnts and Fzds do not participate in endoderm formation. After all, beta-catenin protein is likely to have a much shorter half-life so the fact that MO/RNAi knockdown leads to faster/stronger defects compared to other pathway components would be expected.
The authors detect stronger phenotypes from shWnt3+Fzd5 versus shWnt3, shWnt3+Fzd5 does not seem to be stronger than shFzd5 by itself. This should be stated more explicitly in the text (for example around third sentence, second paragraph p.14).
I agree with the overall conclusion that frizzleds act together for axis patterning, however the extensive combinatorial RNAi presented here seems to reveal further complexity of their possible interactions. For example, Fz1+Fz10 RNAi seems to elicit the expansion of bra phenotype, along more weakly with Fz1+Fz4 RNAi, while Fz4+Fz10 RNAi seems to reduce the bra domain. In addition given the phenotype of Fz5 RNAi alone (less bra and wnt2, more six3/6), some of the double RNAi conditions that resemble controls (eg, Fzd1+5 RNAi, Fzd5+10) might be interpreted as suppression of this phenotype because of opposing gene actions.
These observations could potentially point to a more complex model in which only some of the Fzds signal through lrp5/6 but that canonical Wnt signaling is required for the overall process.
What happens to the expression of the key Wnts and Fzds described here after inhibition of pathway factors? It seems possible that there could be additional complexity to this pathway given the large number of possible ligands/receptors and mutual interactions. For example, the interpretations of the double-RNAi experiments could be modified if for example Wnt4 suppressed Fzd5 expression. Does Wnt4 expression require Wnt3 or perhaps vice versa? I don’t think this is essential for publication but the authors could perhaps point out any existing information of this nature.
I would suggest making a visual cartoon model of the signaling and localization of the components of this model which will would help synthesize the findings for a broader audience.

First revision

Author response to reviewers' comments

Reviewer 1 Advance Summary and Potential Significance to Field:
This is an interesting, well convinced and well written manuscript, providing solid data on the role of the extracellular components of the Wnt Beta catenin signaling pathway during embryogenesis and larval formation in the sea anemone Nematostella vectensis. By performing a comprehensive set of knockdown (KD) experiments, the authors show the respective role of LRP5/6 and Frizzled receptors in patterning the oral aboral axis. They show that the four Nematostella Frizzled receptors are partially redundant. They also convincingly contradict recently published results (Wijesena et al. 2022) on the function of Frizzled 1 and Frizzled 10 during Nematostella embryogenesis. Finally, they address the role of the Wnt ligands in oral aboral patterning and show that double KD Wnt3/Wnt4 is sufficient to phenocopy LRP5/6 or Frizzleds KD. This study is an excellent addition to the literature as it represents the most comprehensive analysis of the role of the Wnt pathway extracellular components performed in a cnidarian.

Reviewer 1 Comments for the Author:
I would suggest a few experiments to strengthen the data and some corrections in the text and figures.

Major comments:

KD efficiency following shRNA injection should be estimated by qPCR. This is particularly relevant

© 2022. Published by The Company of Biologists under the terms of the Creative Commons Attribution License (https://creativecommons.org/licenses/by/4.0/).

for the KD of the Wnt genes as these have not been confirmed by morpholino injection and many Wnt shRNA injections lead to no clear phenotype.
Response: We have performed the qPCR analysis. It is presented on the Supplementary Fig. 2B

It is reported on page 14 that Wnt1 and Wnt3 KD “resulted in a reduction of the expression of the oral marker Bra, and its expansion to the bottom of the pharynx”. I however cannot see this in the images provided in Figure 6. The authors would need to show more convincing data and/or downplay this result.
Response: We replaced the image in question for what we think is a clearer photo and added an extra Supplementary Fig. 10 of the 3 days old planula, where the expansion of *Bra* expression to the bottom of the pharynx is better visible.

The rational for focusing on the role of Wnt3 and Wnt4 for multiple knockdown experiments (“KDs of Wnt3 and Wnt4 resulted in the strongest patterning changes”) is questionable as Wnt3 KD phenotypes are barely noticeable. The authors conclude from the reported Wnt3/Wnt4 KDs that “these two orally expressed Wnt ligands play the main role in the Fz/LRP5/6-mediated OA patterning during early Nematostella development. KD phenotype similarity also suggests that among these two Wnt ligands, Wnt4 appears to be the one predominantly signalling via the aborally expressed Fz5.” (page 17). Without fully addressing the role of the other Wnts, these conclusions seem premature. I would suggest either downplaying the conclusions on the role of Wnt3 and Wnt4 in Fz/LRP5/6-mediated OA patterning or fully investigating the role of the other Wnts (most importantly Wnt2 for which KD results in clear oral expansion of Six3/6) performing double KD experiments with the other Wnts and Frizzleds, as they did for Wnt3 and Wnt4.
Response: We disagree that Wnt3 KD phenotypes are barely noticeable, and we hope that we also manage to convince the Reviewer with a better photo on the Fig. 6A and an additional Supplementary Figure 10. In the original manuscript, we focused on the Wnt3/Wnt4 double KD because individual knockdowns of theses produced strongest phenotypes in our hands. In the revised version of the paper, we also include Wnt/Fz double knockdowns for Wnt1, Wnt2 and Wnt8a (Suppl. Fig. 12). We did not perform double Wnt/Fz KD combinations for other Wnt genes, because we did not observe any phenotypes in the individual knockdowns. We also performed double KDs of all Wnt genes, for which we observed a phenotype at the gastrula stage (Supplementary Fig. 13 and 14). As expected, Wnt1/Wnt4 phenotype was similar, albeit somewhat weaker, to the Wnt3/Wnt4 phenotype.

Minor comments:

Figure 1. How was the picture “Fz10 10hpf” oriented? The staining on the lower part of embryo appears weaker than on the upper part. Considering that Fz10 expression at gastrula stage is restricted to the oral side, shouldn’t we reverse the “Fz10 10hpf” image?
Response: We double-checked this – the picture may be misleading since the future pre-endodermal plate devoid of *Fz10* expression is out of focus, and only the aboral weakening of expression is observed. We replaced it with a better image.

Page 6. In situ hybridizations of Fz1, Fz5 and Fz10 at several developmental stages have been already published in Leclère et al. 2016 and Wijesena et al. 2022. This prior work should be acknowledged when describing Figure 1 in the first section of the results.
Response: We thank the Reviewer for this comment. We made sure that all the relevant references are included.

Page 5. “At the onset of gastrulation, Fz1, Fz4 and LRP5/6 are weakly expressed ubiquitously.” As in situ hybridization is not a quantitative method, this sentence is misleading. Moreoever, according to Supp Figure 1, those three genes do not seem to be particularly lowly expressed at this stage. The word ‘weakly’ should be deleted.
Response: Done

Page 5. “aboral ectoderm appears to be free of Fz1 mRNA.” This cannot be assessed by in situ hybridization. Please rephrase.
Response: We removed this fragment of text.

© 2022. Published by The Company of Biologists under the terms of the Creative Commons Attribution License (https://creativecommons.org/licenses/by/4.0/).

Figure 2. In the triple injection shFz4+5+10, Bra and Wnt2 seem to be expressed on only one side of the blastopore. Is this systematically observed? If not, this should be specified in the figure legend; if it is, this information must be mentioned in the results. Could this be linked to the peculiar morphology of these injected larvae?
Response: Thanks for the comment. This asymmetry, likely reflecting some problems with the directive axis patterning, has also been noticed by the Reviewer 2 (please see a very similar comment below). We frequently observed it but we do not yet know what it means, so we focused on phenotypes related to the oral-aboral axis. The central role of b-catenin signalling in establishing the early radial expression of *BMP2/4* and *Chordin*, as well as the gradual reduction of *Wnt1*, *Wnt3*, *Bra* and *FoxA* expression in BMP2/4 morphants, has been demonstrated before (Kirillova et al., 2018, https://doi.org/10.1073/pnas.1711516115). Thus, problems with the directive axis patterning are not unexpected. Currently, we do not know how oral-aboral and directive axis patterning systems are coordinated. Testing this systematically requires a lot of additional experimentation and will have to be performed in the future as a separate study. We added a note in the legend to the Fig. 2 and edited the text mentioning a possible problem with the interplay between the oral-aboral and the directive axis patterning mechanism in the description of the Fz10MO phenotype.

Page 9 "manifest itself after the RNAi." A word is missing: "RNAi injection"?
Response: "…after the RNAi" should be sufficient. "RNAi injection" does not sound right: it would mean "after RNA interference injection".

Page 11. "Individual Fz RNAi did not lead to significant morphological defects apart from a slight gastrulation delay in Fz1 and Fz10 RNAi, and a previously reported slight shortening of the OA axis in Fz5 RNAi (Leclère et al., 2016)." Please provide more information about these delays. Also, were apical organs reduced in Fz5 shRNA-injected larvae as described for Fz5 MO?
Response: We do not know exactly what kind of additional information we can add here. Fig. 3B shows that shFz1 and shFz10 embryos appear to catch up with controls. We also performed anti-acetylated tubulin antibody staining of shFz5 embryos to address the apical organ effect of the RNAi.

The outcome was quite variable: in some cases, apical organs appeared reduced, as reported in the morphants, in other cases, apical organs appeared to be normally sized. In contrast to morpholinos, shRNAs are not very stable molecules. Since apical organs develop only by 3 dpf, at which point RNAi effect may have substantially declined, the variability of the apical organ phenotypes is not very surprising. Since we cannot properly interpret the outcome of this experiment, and since the early effect of Fz5 RNAi clearly phenocopied the described effect of the Fz5MO on the expression of the oral, midbody, and aboral markers, we do not show the outcome of these antibody stainings in the revised version of the manuscript.

Page 14. "RNAi of Wnt5, which was not very efficient with both shRNAs we used)". A parenthesis is missing.
Response: Thank you for noticing. We replaced a parenthesis with a closing comma.

Supplementary Table 2. Please indicate whether the MO sequences used in this manuscript were new or already published. Notably, is Fz10 MO the same as the recently published one (Wijesena et al. 2022)?
Response: Sequences included, Fz10MO sequence was the same as in Wijesena et al., 2022 – we also added the reference to Wijesena et al., 2022 to the table with the morpholino sequences in the Supplement.

Please indicate in the Material and Methods and/or in the supplementary material the quantity/concentration used for each shRNA, Morpholino and mRNA injections (single, double, triple and quadruple).
Response: We apologize for forgetting to add these important details in the original version. We added the shRNA and the morpholino concentrations and commented on the controls in the Methods section.

Supp Figure 5. Correct AXIN by Axin.
Response: Done

© 2022. Published by The Company of Biologists under the terms of the Creative Commons Attribution License (https://creativecommons.org/licenses/by/4.0/).

Supp Figure 8. Correct dnFz-mCherry with dnFz1-mCherry in the figure legend.
Response: Done

Reviewer 2 Advance Summary and Potential Significance to Field:
Niedermoser et al. provide a neat demonstration of how the canonical Wnt pathway patterns the oral-aboral axis in the cnidarian Nematostella vectensis. The study further shows that cWnt pathway additionally modulates the directive axis, and that endoderm specification is likely dependent on maternal beta-catenin signal. Combinatorial loss-of-function experiments show that the receptor LRP5/6 plays a major role, that the different Frizzled receptors have partially redundant roles, and that the ligands Wnt3 and Wnt4 appear to be particularly relevant for the set up of the oral-aboral axis and the development of endodermal structures. The study is well done, and the images provided are of high quality. Eventual discrepancies with previous data and among different approaches are convincingly addressed.
This study represents a solid and durable contribution to our understanding of body patterning in a relevant cnidarian model.

Reviewer 2 Comments for the Author:
I have one major recommendation, and some further minor comments (in order of appearance).

M1. Given that the phenotypes observed are not independent of the knockdown approach used (shRNA, MOs, dominant negative constructs) and that in some cases no effect was observed, the Authors need to:
- provide further details on the knockdown experiments, in particular regarding the concentrations of shRNA and MO and which controls where used (for example, it appears that a generic control MO was used for all experiments, instead of a gene-specific one; no information is provided on the RNAi control).
Response: We apologize for forgetting to add these important details in the original version. We added the shRNA and the morpholino concentrations and commented on the controls in the Methods section.

- asses independently the efficiency of KDs, as in situ hybridization alone is not quantitative, and stainings can be misleading. This is best exemplified by the case of Wnt2 KD: in supplementary figure 2A we see that the two shRNAs against Wnt2 suppress the expression of Wnt2 itself (with the second shRNA against Wnt2 having a stronger effect), however the same KD would be judged as less efficient when considering only figure 6A. This would allow to further clarify the unsuccessful KD of Wnt5 (judged "not very efficient", page 14) and WntA. I recommend performing a stage-specific quantification of mRNA levels, with qPCR, as already done by the same Authors in Lebedeva et al., 2021.
Response: We provide qPCR testing as a new Supplementary Fig. 2B.

m1: This is largely a matter of personal taste, but I would recommend starting the Introduction section with the explanation of the roles and the evolution of Wnt signaling, and introduce only later the details of the pathway (which non-experts might find harder to follow).
Response: We tried swapping the parts of the introduction and decided on leaving it as it was.

m2. The sentence "a typical beta-catenin loss-of-function phenotype" (page 3) needs a reference.
Response: We inserted the reference

m3. Please move the technical details (e.g. "from cDNA") at the beginning of Results section to the Methods.
Response: We removed the details as requested.

m4. The fact that LRP4/5/6-like displays an anomalous intracellular domain does not necessarily rule out all involvement in cWnt signaling: it could still modulate the signal, for example acting as a dominant negative. Instead, as it is expressed only later in the apical organ region, we can conclude that it does not take part in patterning the OA axis. Please, rephrase.
Response: We agree. We removed the sentence regarding the unconventional intracellular domain.

© 2022. Published by The Company of Biologists under the terms of the Creative Commons Attribution License (https://creativecommons.org/licenses/by/4.0/).

m5. The multiple KD of Fz4, Fz5 and Fz10 shows a defect in the radial expression of Bra and Wnt2 (Figure 2) and the same combinatorial KD at planula stage show a defect in the organization of the mesenteries (Figure 3C). Additionally, MO against Fz10 alone caused a defect in the organization of the mesenteries (Figure 5B) - currently interpreted as a delay (pg.11). These findings could instead point to a role of (some) Fz receptors in setting up the directive axis, together with LRP5/6 (Supplementary Figure 6). Please, comment these data.

Response: Thanks for this comment. We agree. Please see a very similar comment from the Reviewer 1. I am copy-pasting my answer to the Reviewer 1 here (with minor changes):
We frequently observed this asymmetry but we do not yet know what it means, so we focused on phenotypes related to the oral-aboral axis. The central role of b-catenin signalling in establishing the early radial expression of *BMP2/4* and *Chordin*, as well as the gradual reduction of *Wnt1*, *Wnt3*, *Bra* and *FoxA* expression in BMP2/4 morphants, has been demonstrated before (Kirillova et al., 2018, https://doi.org/10.1073/pnas.1711516115). Thus, problems with the directive axis patterning are not unexpected. Currently, we do not know how oral-aboral and directive axis patterning systems are coordinated. Testing this systematically requires a lot of additional experimentation and will have to be performed in the future as a separate study. We added a note in the legend to the Fig. 2 and edited the text mentioning a possible problem with the interplay between the oral-aboral and the directive axis patterning mechanism in the description of the Fz10MO phenotype.

m6. The rationale for focusing on Wnt3 and Wnt4 KDs as they present the "strongest patterning changes" (pg. 14) is, in my opinion, not justified: the changes in patterning for Wnt8a and Wnt2 KDs are also strong, only that they affect the aboral/midbody boundary. Additionally, also Wnt1 KD shows an interesting phenotype in the Bra expression domain. Please, rephrase.
Response: In the new version of the paper, we add the analysis of double KDs of all Wnt genes showing a noticeable phenotype in single KDs at the gastrula stage. Please see Supplementary Figures 13-14.

m7. Please correct "Fig. 6C" (last line of page 14) to "Fig. 6D".
Response: We added a “D” to Fig. 3 in this reference. Fig. 6C, however, was correct.

m8. One of the main conclusions of the manuscript is that Wnt3 and Wnt4 are required for the maintenance of the directive axis. It would be good to provide a phalloidin-stained planula stage, showing the organization of the mesenteries in the case of KD with shWnt3 and shWnt4, separately – in complement of the combinatorial KD.
Response: We provide two new supplementary figures: Suppl. Fig. 11 with phalloidin-stained planulae upon individual KDs of all Wnt genes, which led to a phenotype at the gastrula stage, and Suppl. Fig. 14 with all possible combinations of double knockdowns of these Wnt genes at the same stage.

m9. Some bibliographic references are missing – e.g. when describing the expression dynamics of Fz receptors in Figure 1 and the KD phenotypes (Figure 2). In particular, the paper Lebedeva et al., 2021 is widely cited, but several genes were identified in other studies (eg. Sinigaglia et al., 2013 for Six3/6; Leclere et al., 2016 for Fz5 and Wijesena et al., 2022 for Fz1 and Fz10).
Response: We inserted the references. Thank you for pointing this out.

m10. In all figures:
- I recommend changing the current style of labels (for example) "shFz1 +Fz4 +Fz10" to the more specific "shFz1 + shFz4 + shFz10";

Response: We agree that this way of labelling is better, however, we tried it, and it required reducing the font such that it made the labels poorly readable. We left the old labelling system but added a comment in the figure legend to the first Fig with multiple KDs (Fig. 2), where we specify the labelling convention.

- please specify the developmental stage/number of hours post fertilization in figure legends, for both main manuscript and supplementary section.

© 2022. Published by The Company of Biologists under the terms of the Creative Commons Attribution License (https://creativecommons.org/licenses/by/4.0/).

Response: We specified the stage of the embryos for all figures.

m11. Figure 1: I am confused by the expression of Fz10 (compare 10 hpf and 15 hpf): is the orientation of the embryo correct?
Response: Thanks for pointing this out. The orientation is correct. However, we replaced the 10 hpf image with another one showing a “bald patch” in the *Fz10* expression at the position of the future preendodermal plate. By 15 hpf, the plate starts to express *Fz10*, and this expression is maintained in the gastrula and planula.

m12. Figure 1: The removal of Fz1 expression from the endodermal plate is not clear, in particular when comparing the 10 and 15 hpf stages. I recommend providing a clearer image, if possible.
Response: *Fz1* is a weakly expressed gene with a ubiquitous expression and then near-ubiquitous expression at these stages. We cannot provide an image, where clearing is more apparent, but we are convinced that our observation is correct. We modified the sentence suggesting that Fz1 clears out of the preendodermal plate to make it less definitive.

Reviewer 3 Advance Summary and Potential Significance to Field:
Genikhovich and colleagues present here a functional analysis of the roles for frizzled receptors in control of patterning the primary body axis in Nematostella. The detailed nature of the Wnt signaling mechanisms controlling axis specification in this organism have not yet been fully resolved, in particular the correspondent roles between the several Wnts and Frizzled receptors across the oral-aboral axis in that organism. The study identifies an lrp5/6 factor expressed dynamically in embryogenesis and similar to Fz5 whose expression gradually restricts aborally. lrp5/6 inhibition caused enlargement of ectodermal aboral identity (six3/6) at the expense of oral identities (bra, wnt2) but without causing gastrulation failure or endoderm specification. Knockdown of fzd5 partially phenocopied this effect and inhibition of Fz1+Fz10+Fz4+Fz5 enhanced these phenotypes. The authors then identify Wnt3 and Wnt4 as acting together. Together the study identifies a molecular pathway responsible for the early establishment of the oral/aboral axis. The use of Wnt signals to control polarity of the primary body axis is likely ancient, indicating the importance of understanding the detailed Wnt axis specification mechanisms will be of broad interest to developmental biologists. The study is strongly supported and clearly written. I have only a few minor suggestions below.

Reviewer 3 Comments for the Author:
Minor comments
The authors note some discrepancy between their analysis and another lab’s previously published work regarding Fz1 and Fzd10. However, I find the present study to be compellingly executed and an important addition to the field. In some cases, the discrepancy could also be due to the nature of the perturbations, with dominant negative Fz1 expression potentially able to disrupt other Fz-dependent signaling.

On p.9 it seems possible that a perdurance of LRP5/6 protein, either maternally deposited or already translated in early embryogenesis, could potentially be involved in endoderm specification.
Response: We cannot exclude this, but LRP5/6 is not in the proteomic dataset of Levitan et al., 2015.

The language the authors use is indeed careful (“we do not find evidence of its involvement…”) but a possible explanation of inability to sufficiently reduce any early embryonic LRP5/6 protein should be more fully elaborated in discussion.
Response: We are being extremely careful with the language when discussing this, and we stated already that genetic approaches will be required to definitively answer this question:
“In the future, generation and incrossing of the β-catenin$^{wt/-}$ and LRP5/6$^{wt/-}$ knockout lines will dowus to definitively answer the question whether or not endoderm specification relies on maternal β- catenin and is LRP5/6-independent, as our data seem to suggest at the moment.”
We are not sure how we can be even more careful, but we added an extra sentence into the discussion directly before the one cited above:
“Since currently we cannot fully exclude the possibility that some LRP5/6 and Wnt protein remained undetected in all the proteomic datasets (Levitan et al., 2015), or that its translation

© 2022. Published by The Company of Biologists under the terms of the Creative Commons Attribution License (https://creativecommons.org/licenses/by/4.0/).

from maternal mRNA was not sufficiently suppressed in our KDs, additional genetic work will be required."

In general, I think it is difficult to make evidence of absence arguments from morpholino and RNAi experiments, so this issue is also a possible concern with the model that Wnts and Fzds do not participate in endoderm formation. After all, beta-catenin protein is likely to have a much shorter half-life so the fact that MO/RNAi knockdown leads to faster/stronger defects compared to other pathway components would be expected.
Response: We agree that "lack of effect" arguments are always difficult. Therefore, we are writing that generation of mutants is required in the future to definitively answer this question.

The authors detect stronger phenotypes from shWnt3+Fzd5 versus shWnt3, shWnt3+Fzd5 does not seem to be stronger than shFzd5 by itself. This should be stated more explicitly in the text (for example around third sentence, second paragraph, p.14).
Response: We disagree. Individual KD of Fz5 has very little effect on *Brachyury* expression, and shWnt3+Fz5 has a much stronger effect on *Bra* expression than an individual KD of Wnt3.

I agree with the overall conclusion that frizzleds act together for axis patterning, however the extensive combinatorial RNAi presented here seems to reveal further complexity of their possible interactions. For example, Fz1+Fz10 RNAi seems to elicit the expansion of bra phenotype, along more weakly with Fz1+Fz4 RNAi, while Fz4+Fz10 RNAi seems to reduce the bra domain. In addition, given the phenotype of Fz5 RNAi alone (less bra and wnt2, more six3/6), some of the double RNAi conditions that resemble controls (eg, Fzd1+5 RNAi, Fzd5+10) might be interpreted as suppression of this phenotype because of opposing gene actions. These observations could potentially point to a more complex model in which only some of the Fzds signal through lrp5/6 but that canonical Wnt signaling is required for the overall process.
Response: We agree that there is certainly more complexity to this than what we currently understand. For example, we had an idea that Fz1 might be attenuating the effect of other oral Fz genes. However, currently, we cannot comment on these minor phenotypic changes without performing several more years of research. Therefore, in this paper, we focused on what we think are major phenotypic changes and clear outcomes. The fact that only a combined KD of all Fz receptors phenocopies LRP5/6 suggests, however, that all Fz receptors are likely capable of cWnt signalling.

What happens to the expression of the key Wnts and Fzds described here after inhibition of pathway factors? It seems possible that there could be additional complexity to this pathway given the large number of possible ligands/receptors and mutual interactions. For example, the interpretations of the double-RNAi experiments could be modified if for example Wnt4 suppressed Fzd5 expression. Does Wnt4 expression require Wnt3 or perhaps vice versa? I don't think this is essential for publication but the authors could perhaps point out any existing information of this nature.
Response: Thanks for this comment, we agree in principle. In *Nematostella*, *Wnt3*, *Wnt4* and *Fz5* expression is beta-catenin-dependent, so it is expected that their expression will be changed upon mutual knockdowns. We performed the suggested experiments (see image below), and the results were as expected: *Wnt3*, whose expression is positively regulated by cWnt signalling, was reduced in the outer pharynx, where it is co-expressed with Wnt4 (red dashed line). In contrast, *Fz5*, whose expression is negatively regulated by cWnt signalling, was expanded towards the oral pole (just like *Six3/6*). The expression of *Wnt4*, whose expression is also positively regulated by cWnt signalling, became somewhat weaker, while in the Fz5 KD *Wnt4* showed a slight shift towards the inner pharynx, which may reflect an overall reduction of the beta-catenin signalling intensity. Since the effects of the individual KDs of *Wnt3* and *Wnt4* on the oral-aboral markers are quite distinct, and since this information does not change any of the conclusions of the paper, we do not include it into the revised version of the manuscript.

© 2022. Published by The Company of Biologists under the terms of the Creative Commons Attribution License (https://creativecommons.org/licenses/by/4.0/).

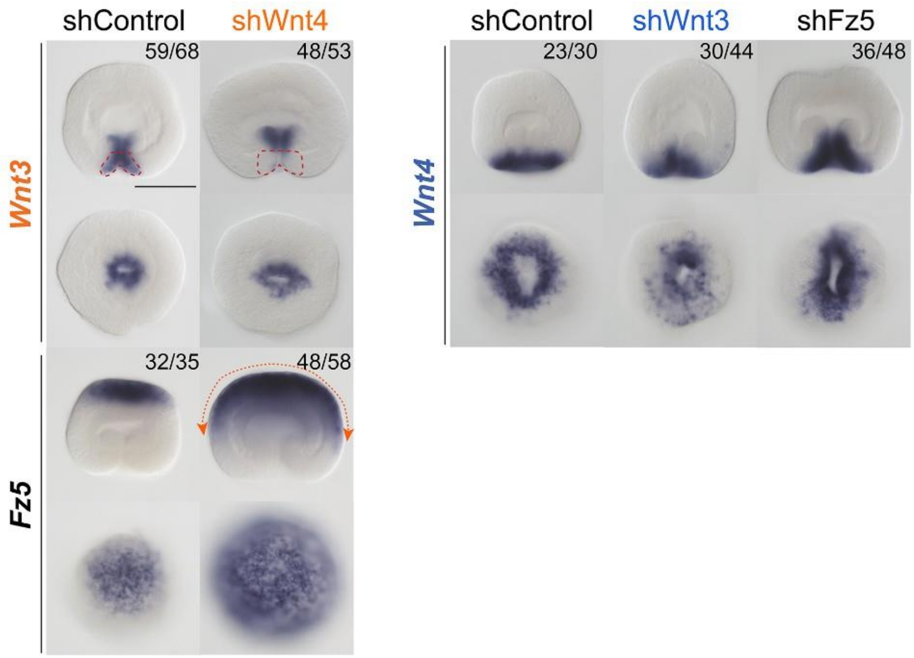

I would suggest making a visual cartoon model of the signaling and localization of the components of this model which will would help synthesize the findings for a broader audience.
Response: Thanks a lot for the suggestion, but we decided against it, since the main outcome of this study is very compact and can be stated in two sentences:
"Taken together, our data suggest the crucial role of the Wnt/LRP5/6/Fz-mediated signaling in the OA patterning of the sea anemone *Nematostella vectensis*, in which different Fz receptors play partially redundant roles. In contrast, we do not find evidence for the involvement of Fz/LRP5/6-mediated signaling in the specification of the preendodermal plate."

Second decision letter

MS ID#: DEVELOP/2022/200785

MS TITLE: Sea anemone Frizzled receptors play partially redundant roles in the oral-aboral axis patterning

AUTHORS: Isabell Niedermoser, Tatiana Lebedeva, and Grigory Genikhovich
ARTICLE TYPE: Research Article

I am happy to tell you that your manuscript has been accepted for publication in Development, pending our standard ethics checks.

Reviewer 1

Advance summary and potential significance to field

The authors did a great job responding to my comments and those of the other reviewers. I have no further comments.

Comments for the author

-

© 2022. Published by The Company of Biologists under the terms of the Creative Commons Attribution License (https://creativecommons.org/licenses/by/4.0/).
